# Hsa-miR-532-3p protects human decidual mesenchymal stem cells from oxidative stress in recurrent spontaneous abortion via targeting KEAP1

**DOI:** 10.1016/j.redox.2025.103508

**Published:** 2025-02-01

**Authors:** Hong Zhou, Jiaxin Zhou, ShanShan Liu, Jing Niu, Jinghua Pan, Ruiman Li

**Affiliations:** a Reproductive Medical Center, The First Affiliated Hospital of Jinan University, 510632, Guangzhou, Guangdong, China; b International School, Jinan University, Guangzhou, Guangdong, 510632, China; c Gynecology Department, Guangdong Women and Children Hospital, Guangzhou, 511442, China; d General Surgery, The First Affiliated Hospital of Jinan University, 510632, Guangzhou, Guangdong, China; e Department of Obstetrics and Gynecology, The First Affiliated Hospital of Jinan University, 510632, Guangzhou, Guangdong, China

**Keywords:** Recurrent spontaneous abortion, Human decidual mesenchymal stem cells, microRNA, Biomarker

## Abstract

**Background:**

Human decidual mesenchymal stem cells (hDMSCs) play crucial roles in pregnancy. The decreased resistance of hDMSCs to oxidative stress is a key factor contributing to recurrent spontaneous abortion (RSA). miRNAs have essential functions in the proliferation and apoptosis of decidual tissues. However, the miRNAs involved in regulating oxidative stress in hDMSCs remain unclear.

**Methods:**

Decidual tissues and hDMSCs were collected from patients with RSA and early pregnancy miscarriages. We assessed the antioxidant capacity of hDMSCs in both groups by detecting relevant indicators. Furthermore, differentially expressed miRNAs in hDMSCs were analyzed through miRNA sequencing. We evaluated the interaction between hsa-miR-532-3p and KEAP1 using a luciferase reporter assay. A mouse model of RSA was constructed for confirmation. Finally, we analyzed the correlations between serum hsa-miR-532-3p levels and the clinical features of pregnant women with RSA.

**Results:**

miRNA sequencing revealed 44 miRNAs whose expression was downregulated and 9 miRNAs whose expression was upregulated in hDMSCs from the RSA group compared with those from the control group. The overexpression of hsa-miR-532-3p led to a significantly increased antioxidant capacity in hDMSCs. The knockdown or overexpression of hsa-miR-532-3p led to the upregulation or downregulation of KEAP1 expression, respectively. In a mouse model, the overexpression of hsa-miR-532-3p reduced embryo absorption rates in RSA mice, decreased KEAP1 expression levels in decidual tissues, and concurrently enhanced the resistance to oxidative stress. Furthermore, in patients diagnosed with RSA, serum hsa-miR-532-3p levels were significantly and negatively correlated with the gestational age.

**Conclusions:**

Our study revealed a lower expression level of hsa-miR-532-3p in the hDMSCs of patients with RSA. Moreover, hsa-miR-532-3p protects hDMSCs from oxidative stress by targeting the Kelch-like ECH-associated protein 1/nuclear factor erythroid 2-related factor 2 (KEAP1/NRF2) pathway. Hsa-miR-532-3p is closely related to gestational age and has good predictive value for identifying RSA.

## Abbreviations

RSA: recurrent spontaneous abortion
MSCs
Mesenchymal stem cells
hDMSCs: human decidual mesenchymal stem cells
ROR: reactive oxygen radicals
OR: oxidative reactions
OS: oxidative stress
miRNAs: microRNAs
T-AOC: total antioxidant capacity
SOD: superoxide dismutase
GSH-PX: glutathione peroxidase
CAT: catalase
IC50: half-maximal inhibitory concentration
GO: Gene Ontology
KEGG: Kyoto Encyclopedia of Genes and Genomes
cDNA: complementary DNA
qPCR: Quantitative real-time polymerase chain reaction
NRF2: NF-E2-related factor 2
KEAP1: Kelch-like ECH-associated protein 1
FBS: fetal bovine serum
PBS: phosphate-buffered saline
EDTA: ethylenediaminetetraacetic acid
NSCLC: non-small cell lung cancer
HO-1: heme oxygenase-1
tBHQ: tert-butylhydroquinone
Maf: muscle aponeurotic fibrosarcoma
AREs: antioxidant response elements

## 1 Introduction

Spontaneous abortion, a prevalent complication of pregnancy, affects a substantial proportion of women attempting to conceive. According to recent data, approximately 15–20 % of diagnosed pregnancies culminate in spontaneous abortion [1,2]. Recurrent spontaneous abortion (RSA) is defined as the occurrence of two or more consecutive spontaneous miscarriages before 20 weeks of gestation and occurs in approximately 5 % of reproductively active couples [3]. The risk of RSA increases incrementally, reaching approximately 24 % following two successive clinical pregnancy miscarriages, 30 % following three miscarriages, and 40 % following four consecutive spontaneous abortions [4]. Unfortunately, our understanding of the complex pathogenesis of RSA remains elusive. Although several possible factors, including environmental, psychological, anatomical, and genetic factors, have been identified, the pathological mechanism of RSA remains unclear [[5], [6], [7]].

Stem cells are a versatile population of precursor cells capable of differentiating into various cell lineages, including osteoblasts, adipocytes, and chondroblasts [8]. Human decidual mesenchymal stem cells (hDMSCs) play a pivotal role in orchestrating the intricate formation of branching villous structures within placental tissues [9]. Moreover, they are essential contributors to the maintenance of placental functionality and facilitate normal fetal development during the early stages of pregnancy [10]. Emerging evidence suggests a correlation between hDMSCs and RSA [11,12]. An analysis of decidual tissue from patients revealed a notable reduction in the vascular density, indicative of the diminished presence of hDMSCs, compared with healthy early pregnancy cohorts [13]. These findings underscore the critical involvement of hDMSCs in fostering vascular development and establishing the maternal–fetal interface. However, the precise association and underlying mechanisms linking hDMSCs to RSA pathology remain unclear.

Many organisms undergo metabolic and physiological processes resulting in the formation of reactive oxygen species (ROS). ROS are vital in cellular processes, and their dysregulation contributes to various pathologies, such as ischemia-related diseases, neurodegenerative disorders, malignant transformation, and aging [[14], [15], [16]]. In addition, excessive ROS accumulation and reduced antioxidant function at the maternal‒fetal interface result in lipid peroxidation, disrupt decidual tissue integrity, and potentially lead to miscarriage [17,18]. Oxidative stress (OS) occurs when the equilibrium between ROS production and intrinsic antioxidant mechanisms is disturbed or when ROS levels exceed the physiological threshold. The antioxidant system refers to the mechanisms and processes that protect cells from damage caused by oxidative stress, which occurs when an imbalance exists between free radicals and antioxidants in the body. OS can induce many pathological consequences and nonselective damage to biological molecules such as proteins, lipids, and DNA, impairing their functions and potentially triggering cellular apoptosis or necrosis [19]. In addition, OS amplification is related to various reproductive diseases, such as polycystic ovary syndrome, endometriosis, and preeclampsia [[20], [21], [22]]. Some studies have implicated OS as a contributing factor to idiopathic RSA [23,24]. Moreover, placental villous tissue experiences a surge in oxygen tension during early pregnancy, from below 20 mmHg at 8 weeks to above 50 mmHg at 12 weeks, leading to OS amplification [25]. Both systemic and placental OS contribute significantly to the pathophysiological manifestations of RSA [26].

MicroRNAs (miRNAs) are small endogenous RNA molecules that modulate gene expression at the posttranscriptional level and are 19–25 nucleotides long [27]. miRNAs play a critical role by repressing the expression of their targets and inducing essential alterations in gene expression programs that underlie various biological processes, including developmental timing, differentiation, proliferation, cell death, and metabolism. Multiple miRNAs have been found to be closely associated with OS, and some of them can inhibit the signaling pathways involved in antioxidant defense pathways, participating in the biological processes that protect cells from OS damage [28]. Notably, scientific studies have substantiated the existence of tissue-specific miRNA expression and the conspicuous abundance of miRNA expression in the human placenta [29]. miRNAs also play roles in placental development and OS resistance through key processes such as antioxidant enzyme expression [30]. Many miRNAs are closely associated with recurrent abortion and play essential roles in regulating the antioxidant system [31]. miRNA-17 and miRNA-19a are significantly downregulated in placental tissues associated with early spontaneous miscarriage, whereas miR-184 is upregulated in the villi and decidua of patients with RSA [32,33]. However, whether a close association exists between the interaction of OS, miRNAs, and RSA, or their intrinsic underlying mechanisms, is still under investigation. NRF2 (nuclear factor erythroid 2-related factor 2) is a cap'n'collar basic-region leucine zipper transcription factor that serves as a central regulator of cellular responses to environmental stresses by inducing the expression of detoxification and antioxidant enzymes, thereby playing a crucial role in defending against oxidative damage [34,35]. KEAP1 (Kelch-like ECH-associated protein 1), a sensor of oxidative and electrophilic stresses, plays a role in negatively regulating the activity of NRF2 [36]. Under oxidative stimuli, ROS bind to the cysteine residues of KEAP1, resulting in a conformational change that inhibits NRF2 ubiquitination and facilitates its translocation into the nucleus [37]. NRF2 then binds to the antioxidant response elements (AREs) in the promoters of antioxidant genes, inducing their transcription [38]. Consequently, NRF2 signaling can modulate numerous antioxidant enzymes; therefore, proper modulation of the NRF2 signaling pathway could ameliorate various cellular responses under OS. However, research on a potential linkage with RSA still lacking.

Thus, in the present study, we screened differentially expressed miRNAs in hDMSCs from RSA patients and found that hsa-miR-532-3p could help antagonize OS-induced damage in hDMSCs. Moreover, we found that hsa-miR-532-3p protected against OS by targeting the KEAP1/NRF2 pathway both in vivo and in vitro. The expression level of hsa-miR-532-3p in the peripheral serum was closely related to the gestational age of RSA patients and had good predictive value for identifying RSA. The findings of this study provide a potential target for the diagnosis and treatment of RSA.

## 2 Materials and methods

### 2.1 Patient selection

Human decidual tissues from patients with RSA and women with primary miscarriage during early pregnancy were collected from January 2021 to December 2022 at The First Affiliated Hospital of Jinan University. Peripheral blood samples were collected between September 2021 and December 2023 at The First Affiliated Hospital of Jinan University and Guangdong Maternal and Child Health Hospital. Patients with RSA were classified into the RSA group, and women with primary miscarriage during early pregnancy were categorized into the control group. This study was approved by the Ethics Committee of the First Affiliated Hospital of Jinan University and conducted in accordance with the Declaration of Helsinki. Informed consent was obtained from all the study participants.

The inclusion criteria for RSA patients were as follows [1]: married females who had experienced two or more natural miscarriages with the same spouse [2]; participants aged over 20 years and under 40 years [3]; miscarriage chorionic karyotypes were found to be normal through a chromosomal karyotype analysis [4]; no anatomical abnormalities were observed in the reproductive system through the detection of gynecological and B-ultrasound examinations [5]; no oral or vaginal medications were used within the past three months [6]; no previous successful pregnancy or parturition history.

The inclusion criteria for women with primary miscarriage during early pregnancy (control group) were as follows [1]: married women with no history of spontaneous abortion [2]; aged >20 years and less than 40 years [3]; no adverse pregnancy history [4]; no abnormal anatomical development of the reproductive system found through obstetric examination and B-ultrasound examination [5]; no oral or vaginal medications used in the past 3 months [6]; gestational weeks 7–11; and [7] no previous successful pregnancy or parturition history.

The exclusion criteria for both groups were as follows [1]: participants with a history of or who were diagnosed with anatomical structural abnormalities of the reproductive system [2]; participants with comorbid endocrine diseases, including diabetes and thyroid diseases [3]; participants with a history of thrombotic conditions and antiphospholipid syndrome [4]; participants with systemic diseases and any malignant tumors [5]; participants with organic or functional diseases or severe mental and psychological disorders [6]; participants who experienced miscarriage caused by nonartificial factors such as work-related fatigue, abdominal impact, or intense physical exercise; and [7] participants with a successful pregnancy or parturition history.

### 2.2 Sample collection and extraction of hDMSCs

Samples were collected from the patients in the RSA and control groups after induced abortion. A total of 5 RSA and 5 normal early pregnant decidual tissues from different individuals were collected to extract hDMSCs. Under aseptic conditions, the chorioamniotic membranes were rinsed with phosphate-buffered saline (PBS, G4202-100 ML, Servicebio, Wuhan, China) containing 100 U/mL penicillin and 100 μg/mL streptomycin (15140122, Gibco™, USA) to remove residual blood. The membranes were then dissected into 1 mm × 1 mm sections and placed in 10 cm culture dishes. Next, 5 mL of digestion solution (0.25 % trypsin-EDTA (25200056, Gibco™, USA) and 0.1 % type II collagenase (C2-28-100 MG, Merck, Germany)) was added, and the tissues were digested at room temperature on a shaker for 90 min. The digestion solution was sequentially filtered through 100, 70, and 40 μm sieves to collect the cells. The collected cells were resuspended in 1 mL of complete DMEM/F12 (11320033, Gibco_TM_, USA; DMEM/F12 supplemented with 10 % FBS, 100 U/mL penicillin, and 100 μg/mL streptomycin) and seeded at a density of 200 cells/mL in 10 cm culture dishes. The dishes were then incubated at 37 °C in a 5 % CO_2_ humidified cell culture incubator for 14 days. Clonally growing adherent cells were harvested and transferred to 24-well plates. Upon reaching 80–90 % confluence, the cells were transferred into 25 cm^2^ cell culture flasks. When the cells reached approximately 85 % confluence, they were passaged at a 1:2 ratio in complete DMEM/F12. The medium was changed every 3 days, and the passaged cells were labeled P1. Subsequent passages were labeled P2, and the cells were cultured until the fourth generation for morphological observation and cell surface antigen detection [39]. The 4th to 9th generations of cells were used for subsequent experiments.

### 2.3 Cell viability detection

The viability of hDMSCs was assessed using the Cell Counting Kit 8 (CCK8) assay (C0038, Beyotime, China). hDMSCs in the logarithmic growth phase were harvested and inoculated in 96-well plates, with 1 × 10^3^ HDMSCs per well. For the oxidative stress resistance test, each group was pretreated with H2O2 at concentrations ranging from 100 to 1000 μM for 24 h. The same fresh medium was used as the negative control. The absorbance was measured at a wavelength of 450 nm was using a Thermo Scientific™ Multiskan™ FC microplate reader. The absorbance values were calculated to evaluate the daily growth activity of the cells after seeding. Each test was performed using at least three independent biological replicates.

### 2.4 Verification of hDMSCs

hDMSCs in the logarithmic growth phase were harvested and inoculated in 6-well plates, with each well containing 5 × 10^5^ cells. Crystal violet (1 %) staining was performed to examine the morphology of the cells. The following markers of hDMSCs were detected by flow cytometry (FC) (BDLSRFortessa): CD3-FITC (1:100, Southern Biotechnology, 9515-02S), CD29-FITC(1:100, Miltenyi, 130-123-692), CD34-FITC(1:100, Miltenyi, 130-113-740), CD44-FITC(1:100, Miltenyi, 130-124-856), CD45-FITC(1:100, Miltenyi, 130-110-631), CD73-FITC (1:100, TRAN, HF101-01), CD90-FITC (1:100, Miltenyi, 130-114-901), and CD105-FITC (1:100, Miltenyi, 130-112-169). The percentage of FITC-labeled cells among the total cell population was determined. The isotype IgG control antibody was used as a negative control.

### 2.5 ROS and antioxidant capability tests

hDMSCs were harvested and inoculated in 6-well plates, with each well containing 5 × 10^5^ cells. ROS levels were detected in the absence of H2O2 (400 μmol) for 24 h, and fresh medium was used as a control. The fluorescent probe DrCFH-DA (D6470, Solarbio, Beijing, China) was used to detect ROS levels in hDMSCs using FC. Additionally, total antioxidant capacity (T-AOC, BC1310-50T, Solarbio, Beijing, China), superoxide dismutase (SOD, BC0175-100T, Solarbio, Beijing, China), glutathione peroxidase (GSH-PX, BC1190-50T, Solarbio, Beijing, China), and catalase (CAT, BC0200-50T, Solarbio, Beijing, China) assay kits and a glutathione disulfide (GSSG)/glutathione (GSH) quantification kit (CAT, G263, DOJINDO, Shanghai, China) were used to measure the total antioxidant capacity. The assessment of the antioxidant capacity was conducted within 8 h, allowing the procurement of human decidual tissue for detection purposes. The antioxidant capabilities of hDMSCs from passages 4 to 9 were subsequently evaluated. The cells were then collected in centrifuge tubes. Following a ratio of cell to extraction liquid volume (mL) ranging from 500 to 1000:1, 1.0 mL of precooled extraction buffer (5 million cells, added to 1 mL of precooled extraction buffer) was added. The cells were subsequently subjected to ultrasonic disruption (at 200 W, with ultrasound on for 3 s and off for 9 s, for a total duration of 3 min). The resulting lysate was centrifuged at 10,000 rpm for 10 min at 4 °C, and the supernatant was collected and stored on ice for further analysis. Each test was performed using at least three independent biological replicates.

### 2.6 Apoptosis and cytotoxicity detection

hDMSCs were harvested and inoculated in 6-well plates, with each well containing 5 × 10^5^ cells. ROS levels were detected in the absence of H2O2 (400 μmol) for 24 h, and fresh medium was used as a control. Apoptosis was assessed by flow cytometry using an ANNEXIN V-FITC/PI apoptosis detection kit (Solarbio, CA1020, Beijing, China). The protocol for the kit was as follows: The cell suspension was centrifuged at approximately 1000 rpm for 5 min to pellet the cells. If certain cells failed to completely pellet, the centrifugation time or force was adjusted accordingly. The supernatant was carefully aspirated, leaving approximately 50 μl of residual culture medium to avoid disturbing the cell pellet. The cells were resuspended in approximately 1 ml of prechilled PBS and centrifuged again to pellet the cells, and the supernatant was gently aspirated. The cell pellet was resuspended in the buffer provided in the kit, and 100 μl of the cell suspension was transferred to a 5 ml flow cytometry tube. After mixing with 5 μl of Annexin V/FITC, the cells were incubated at room temperature in the dark for 5 min, followed by the addition of 5 μl of propidium iodide (PI) and 400 μl of PBS. A flow cytometry analysis was performed promptly. The cell samples were fixed with 4 % paraformaldehyde for terminal deoxynucleotidyl transferase dUTP nick-end labeling to detect apoptosis. The samples were collected after the incubation was complete and washed with PBS to remove the fixative. The TUNEL reaction mixture was prepared according to the instructions of the TUNEL Cell Apoptosis Assay Kit (Solarbio, T2196). The prepared TUNEL reaction mixture was then added to the fixed cell samples to allow the TUNEL reagent to bind to DNA in the cell nuclei. After washes with PBS, the cell nuclei were stained with the fluorescent dye DAPI. Stained cell samples were observed under a fluorescence microscope to evaluate the number of TUNEL-positive cells, and ImageJ (version 1.46r) was used to analyze the distribution of TUNEL-positive cells. Each test was performed using three independent biological replicates.

### 2.7 hDMSC differentiation test

P3 posterior wall cells were cultured to assess whether hsa-miR-532-3p can protect against the oxidative stimulation of differentiation in vitro. hDMSCs were harvested and inoculated in 6-well plates, with each well containing 5 × 10^5^ cells. Oil Red O staining (#C0157S, Beyotime, Shanghai, China), Alizarin Red staining (#C0148S, Beyotime, Shanghai, China), and Alcian blue staining (#G1560, Solarbio, Beijing, China) were performed according to the manufacturer's instructions. For Oil Red O, Alizarin Red and Alcian blue staining, after 10 min of fixation with 4 % paraformaldehyde, the cell samples were incubated with the working solution for 30 min. Images were subsequently captured using an inverted microscope (Olympus 1MT‐2–21, Olympus Corporation, Tokyo, Japan). Each test was performed using three independent biological replicates.

### 2.8 miRNA sequencing

Three sets of hDMSCs from the RSA and control groups were collected for miRNA sequencing. Total RNA was extracted and purified using TRIzol. RNA integrity was evaluated by nondenaturing agarose gel electrophoresis, and qualified RNA samples were used for subsequent high-throughput miRNA sequencing. Sequencing and bioinformatics analyses were conducted by Lianchuan Bio according to the standard procedures provided by Illumina, including library preparation and sequencing experiments. A TruSeq Small RNA Sample Prep Kit (Illumina, San Diego, CA, USA) was used to prepare a small RNA sequencing library.

The sequence quality was verified using FastQC (http://www.bioinformatics.babraham.ac.uk/projects/fastqc). The raw reads were subjected to an in-house program, ACGT101-miR (v4.2, LC Sciences, Houston, Texas, USA), to remove adapter dimers, junk, low complexity, common RNA families (rRNAs, tRNAs, snRNAs, snoRNAs) and repeats. Subsequently, unique sequences with lengths of 18–26 nucleotides were mapped to specific species precursors in miRBase 22.0 by a BLAST search to identify known miRNAs and novel 3p- and 5p-derived miRNAs. Length variation at both the 3′ and 5’ ends and one mismatch inside the sequence were allowed in the alignment. The unique sequences mapped to specific species of mature miRNAs in hairpin arms were identified as known miRNAs. The unique sequences mapped to the other arm of the known specific species precursor hairpin opposite to the annotated mature miRNA-containing arm were considered novel 5p- or 3p-derived miRNA candidates. The remaining sequences were mapped to other selected species precursors (with the exclusion of specific species) in miRBase 22.0 by a BLAST search, and the mapped pre-miRNAs were further BLAST searched against the specific species genomes to determine their genomic locations. The above two miRNAs were defined as known miRNAs. The unmapped sequences were BLAST searched against specific genomes, and the hairpin RNA structures containing these sequences were predicated from the flanking 80 nt sequences using RNAfold software (http://rna.tbi.univie.ac. at/cgi-bin/RNAfold.cgi). The criteria for the secondary structure prediction were as follows [1]: number of nucleotides in one bulge in the stem ≤12 [2], number of base pairs in the stem region of the predicted hairpin ≥16 [3], cutoff of free energy (kCal/mol) ≤ -15 [4], length of the hairpin (up and down stems + terminal loop) ≥50 [5], length of the hairpin loop ≤20 [6], number of nucleotides in one bulge in the mature region ≤8 [7], number of biased errors in one bulge in the mature region ≤4 [8], number of biased bulges in the mature region ≤2 [9], number of errors in the mature region ≤7 [10], number of base pairs in the mature region of the predicted hairpin ≥12, and [11] percentage of the mature region in the stem ≥80.

Analysis of Differential expressed miRNAs: Differential expression (DE) of miRNAs based on normalized deep-sequencing counts was analyzed by selectively using T-Test based on the experiments design. As The abundance and quantity of miRNA are small, and the difference results obtained by FDR value are significant, but some meaningful molecules will be lost. Consequently, we opt to use the original *p*-values for selection. Followed by heatmap analysis and initial screening using volcano plots (DE miRNA screening condition: *P* < 0.05).

Target genes were predicted using TargetScan (5.0, http://www.targetscan.org/) and miRanda (3.3a, http://www.microrna.org) software, and the intersections of the predicted results were selected. Hierarchical clustering analysis was conducted using R (v3.6.0) software based on the differentially expressed miRNAs. A Gene Ontology (GO) functional enrichment analysis was performed using hypergeometric tests to identify GO terms significantly enriched in target genes corresponding to differentially expressed miRNAs, thereby determining the associated biological functions. A Kyoto Encyclopedia of Genes and Genomes (KEGG) pathway enrichment analysis was performed to identify significantly enriched pathways of the target genes.

### 2.9 Cell transfection

For miRNA transfection, 5 × 10^5^ hDMSCs were cultured in 6-well plates in culture medium in a humidified incubator at 37 °C with 5 % CO2. The transfection complex was prepared by mixing miRNA mimics with the transfection reagent (Lipofectamine™ RNAiMAX, Invitrogen™, 13778075, USA). The miRNA–Lipofectamine® RNAiMAX complexes were prepared as follows: 3 μl (30 pmol) of miRNA (negative control (NC) mimic, hsa-miR-532-3p mimic, or hsa-miR-532-3p inhibitor) was diluted in 150 μl of Opti-MEM® I reduced serum medium (31985062, Gibco™, USA). In addition, 9 μl of Lipofectamine® RNAiMAX was diluted in 150 μl of Opti-MEM. The complexes were then mixed gently and incubated for 5 min at room temperature. For each well to be transfected, the medium in each well was replaced with 1.75 ml of fresh culture medium without antibiotics. A total of 250 μl of the complex was then added to each well and incubated for 24 h at 37 °C with 5 % CO2. The culture medium was then changed for further analysis. The sequences of the miRNA mimics and negative control are shown in Supplementary Table 1.

For the cotransfection of miRNA and the DNA vector (negative control (NC) vector, hsa-miR-532-3p mimic, hsa-miR-532-3p inhibitor and KEAP1 expression plasmid), Lipofectamine 2000 (Invitrogen™, 11668027, USA) was used. One day before transfection, 5 × 10^5^ hDMSCs were cultured in 6-well plates with growth medium without antibiotics. For each transfected sample, the following DNA‒miRNA molecule‒Lipofectamine 2000 complexes were prepared: 500 ng of DNA and 30 pmol of miRNA in 250 μl of Opti-MEM and 6 μl of Lipofectamine 2000 in 250 μl of Opti-MEM. The mixture was mixed gently and incubated for 5 min at room temperature. Five hundred microliters of the complexes was added to each well containing 1.5 ml of fresh medium. The cells were incubated at 37 °C in a CO2 incubator for 24 h, after which the culture medium was changed for further analysis.

### 2.10 Dual-luciferase reporter assay

The Dual-Luciferase® Reporter analysis system (E1910, Promega, Beijing, China) was used to detect dual-luciferase reporter genes. Plasmids carrying the wild-type and mutant KEAP1 sequences were constructed to assess the interactions between the predicted miRNAs and specific potential targets. The predicted miRNA mimics and the wild-type and KEAP1 mutant plasmids or negative control plasmids were cotransfected into hDMSCs. Renilla luciferase activity was used as a control to normalize luciferase activity. Each test was performed using three independent biological replicates.

### 2.11 RT‒qPCR

Total RNA was extracted from the samples using TRIzol reagent (Invitrogen, 15-596-018, Carlsbad, CA, USA), and the concentration of RNA was determined using a NanoDrop 2000 (Thermo Scientific, Waltham, MA, USA). The extracted RNA was reverse transcribed into complementary DNA (cDNA) using an RT First Strand cDNA Synthesis Kit (Service Bio, Wuhan, China). Quantitative real-time polymerase chain reaction (qPCR) was performed with a CFX RT‒PCR system (Bio-Rad Laboratories, Inc.) using SYBR Green qPCR Master Mix (K0252, Invitrogen, USA). U6 was used as the internal reference gene to normalize the expression levels of the miRNAs, whereas GAPDH served as the internal reference gene for the other target genes. The sequences are listed in Supplementary Table 2. Each test was performed using three independent biological replicates.

### 2.12 RNA immunoprecipitation (RIP)-qPCR

An RNA-binding protein immunoprecipitation kit (Millipore Sigma, Burlington, MA, USA) was used to determine the relationship between *KEAP1* and hsa-miR-532-3p according to the manufacturer's protocol. Anti-argonaute 2 (Ago2) (SAB4200085, Millipore Sigma, Burlington, MA, USA) and control immunoglobulin G (IgG) (I4506, Millipore Sigma, Burlington, MA, USA) antibodies were used to perform the RIP assays, and the expression levels of KEAP1 and hsa-miR-532-3p were subsequently evaluated using qPCR. The protein precipitation complex levels of Ago2 were analyzed by western blotting. Each test was performed using three independent biological replicates.

### 2.13 Western blot

Cell lysates were prepared using RIPA lysis buffer (Thermo Fisher Scientific, 89900) supplemented with a protease inhibitor cocktail. The protein concentration was determined using the BCA method. Protein samples were separated on 10–12 % SDS‒PAGE gels and transferred onto nitrocellulose membranes. The membranes were then blocked with 5 % skim milk and incubated overnight with primary antibodies, including anti-GDF11 (1:1000, rabbit polyclonal antibody #10503-2-AP, Proteintech, USA), anti-NRF2 (1:1000, rabbit polyclonal antibody #16396-1-AP, Proteintech, USA), and anti-GPX4 (1:1000, rabbit polyclonal antibody #14432-1-AP, Proteintech, USA) antibodies, with β-actin (1:2000, ab6276, Abcam, Cambridge, UK) serving as an internal control. After three washes with TBST solution, the membranes were then incubated with secondary goat anti-rabbit IgG antibodies conjugated with horseradish peroxidase (1:2000, ab109489, Abcam, Cambridge, UK) at 37 °C for 1 h. Subsequently, the membranes were immersed in an electrochemiluminescence solution for imaging, followed by an analysis of relative protein levels using an imaging system.

### 2.14 Animal models

All animal experiments received ethical approval from the Laboratory Animal Ethics Committee of Jinan University (IACUC-20201126-05) and were performed according to the ARRIVE guidelines. The sample size was based on previous experiments, and the experimental unit was a single animal. The investigators who conducted the injections and outcome assessments were blinded to the group allocations. Thirty female CBA/J mice were randomly divided into three groups. Twenty female CBA/J mice were cohoused with five male DBA/2J mice to establish a miscarriage model and further divided into a miRNA intervention group and a negative control group. Another 10 female CBA/J mice were cohoused with 5 male BALB/c mice to establish a normal pregnancy model, which served as the normal control group. The three groups of female mice were housed under the same conditions. The specific method involved housing the three groups of female mice; within the first 4 days of cohabitation, no copulatory or suspicious plugs were detected within the first four days of cohabitation. Two days later, the male mice were exchanged for a second round of cohabitation. The day when thrombi were detected was considered Day 0.5 of pregnancy. Following the second cohabitation, the pregnancy stage was estimated based on suspicious copulatory plugs and changes in body weight. The abortion rates of the two groups were calculated as follows: abortion rate (%) = [number of absorbed mutants/(number of absorbed resonances + number of surviving mice)] × 100. Vaginal plugs were checked every morning to determine whether the mice had mated. The day on which a vaginal plug was observed was marked as Day 0 of pregnancy [40]. Agomir NC or agomiR-532-3p (MIXSUNGEN, Shanghai, China) was injected into mice with normal pregnancies (NP group). The RSA mice were randomly divided into two groups: an RSA agomir NC group, which consisted of mice injected with agomir-NC, and an RSA agomiR-532-3p group, which consisted of mice injected with agomiR-532-3p (RiboBio, Guangzhou, China). All mice were injected via tail at the dose of 10 nmol of agomir-NC or agomiR-532-3p per mouse in 200 μl of saline respectively [7,41]. Starting on Day 0, all the mice received intravenous injections of a saline solution every 3 days. The mice were sacrificed on Day 11.5, and the embryo resorption rate was calculated. Uterine decidual tissues were collected for western blotting, qRT‒PCR, and flow cytometry. Nonpregnant mice were excluded, and ten mice from each group were selected for further analysis. HE staining was performed to examine the morphology of the decidual tissue of pregnant mice, and immunohistochemistry (IHC) was performed to assess the expression levels of the target protein KEAP1 within the decidual tissue of pregnant mice. The expression levels of OS-related markers and hDMSCs were compared.

### 2.15 Immunohistochemistry (IHC)

IHC was performed on decidual tissue sections to examine the expression of specific proteins. The tissue samples were fixed with 10 % neutral buffered formalin and embedded in paraffin. Thin sections (4 μm) were cut using a microtome and placed on glass slides, which were dried overnight at 37 °C. Deparaffinization was performed with xylene, followed by rehydration through a series of graded ethanol solutions. Antigen retrieval was accomplished by immersing the sections in citrate buffer (pH 6.0) and heating them in a microwave for 15 min. After cooling, the sections were rinsed with PBS and incubated with 3 % hydrogen peroxide to block endogenous peroxidase activity. Blocking was performed for 1 h with 5 % bovine serum albumin (BSA) in PBS. The sections were then incubated overnight at 4 °C with primary antibodies (anti-KEAP1, 1:200, ab218815, Abcam, Cambridge, UK) specific to the target protein. After washes with PBS, secondary antibodies conjugated with horseradish peroxidase (HRP) were applied and incubated with the sections for 1 h at room temperature. The peroxidase activity was visualized using 3,3′-diaminobenzidine (DAB, DA1010, Solarbio, Beijing, China) substrate, and the tissues were counterstained with hematoxylin. The sections were then dehydrated, cleared, and mounted for the microscopic analysis. IHC scores were calculated according to the proportion of positive cells; none, 1–25 %, 26–50 %, 51–75 %, and 76–100 % were calculated as 0–4 points, respectively, and based on the staining intensity, the cells with no, weak, medium, and strong staining were calculated as 0–3 points, respectively. Finally, the two scores were added to obtain a comprehensive score.

### 2.16 Statistical analysis

Normally distributed data are presented as the means ± standard deviations. The authors assessed the distributions of the data using statistical methods such as the Shapiro‒Wilk test or Kolmogorov‒Smirnov test to evaluate whether the data followed a normal distribution. These tests compare the observed data distribution with a theoretical normal distribution. If the P value obtained from these tests was greater than the significance level (usually set at 0.05), the data were normally distributed. Graphical methods such as histograms, Q‒Q plots, and density plots were also used to visually assess the normality of the data distribution. A *t*-test was used for comparisons between two groups, whereas one-way ANOVA and SNK tests were used for multiple comparisons. Nonnormally distributed data are presented as medians and interquartile ranges, and nonparametric tests were applied. Spearman's correlation analysis was used to assess the correlations. Differences in quantitative and qualitative data between groups were compared using t tests and chi-square tests, respectively. The diagnostic value of hsa-miR-532-3p was evaluated by constructing a receiver operating characteristic (ROC) curve. When the expression level of hsa-miR-532-3p reached the maximum area under the ROC curve (AUC), the best cutoff value for predicting RSA diagnosis was determined, and the sensitivity and specificity were evaluated. A P value < 0.05 was considered statistically significant. All the data were processed using R software and GraphPad Prism 7.0.

## 3 Results

### 3.1 Extraction and identification of hDMSCs

**Fig. 1:**
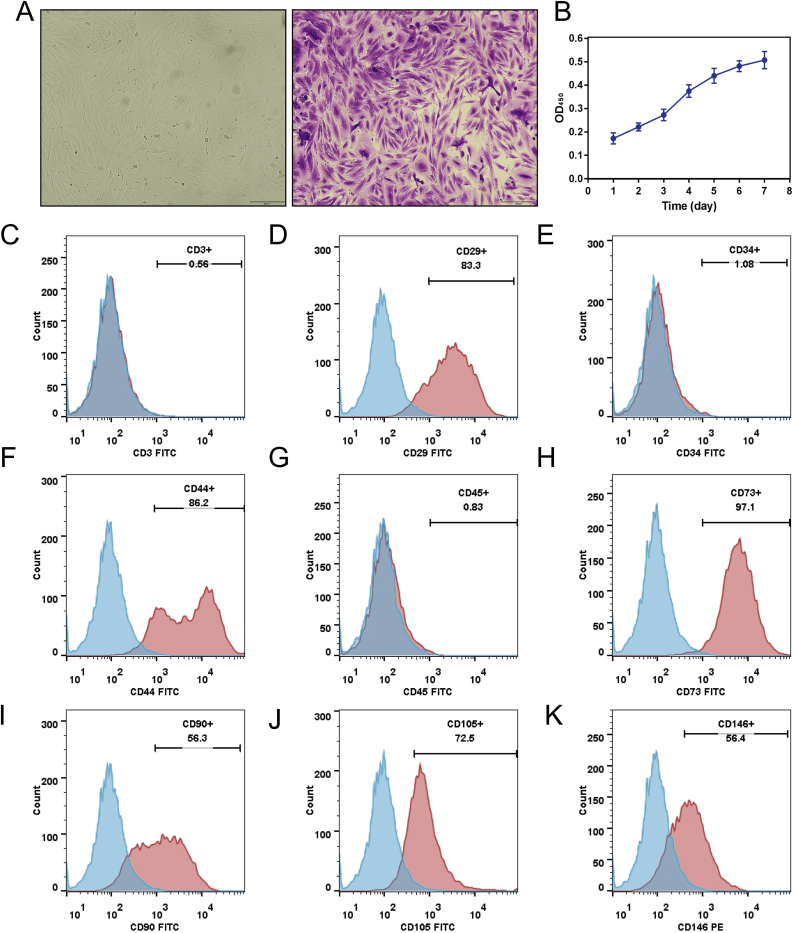
Extraction and identification of hDMSCs. A. The morphology of early pregnancy hDMSCs was observed under brightfield microscopy and crystal violet staining. B. The growth curve of early pregnancy hDMSCs was determined using CCK8 assay (n = 5), data representing mean ± SD. C–K. Flow cytometry was used to evaluate the expression of cell markers CD3, CD29, CD34, CD44, CD45, CD73, CD90, CD105, and CD146 in early pregnancy hDMSCs.

**Supplementary Fig. 1:**
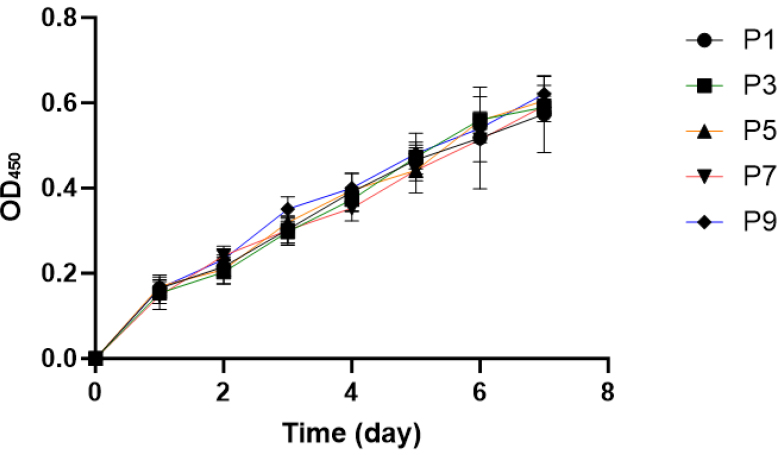
The growth curve of different passage hDMSCs was determined using CCK8 assay (n = 5), data represent mean ± SD.

**Supplementary Fig. 2:**
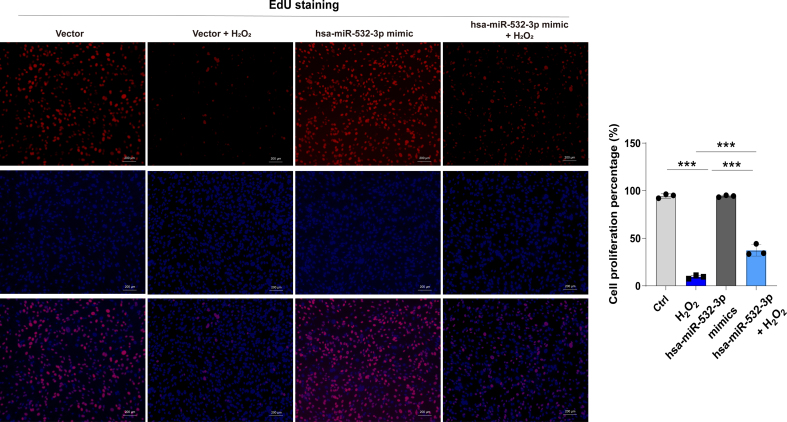
Changes in cell proliferation of hDMSCs transfected with hsa-miR-532-3p mimic under H_2_O_2_ intervention detected by EdU staining (n = 3). Data represent mean ± SD, ∗∗∗*P* < 0.001.

**Supplementary Fig. 3:**
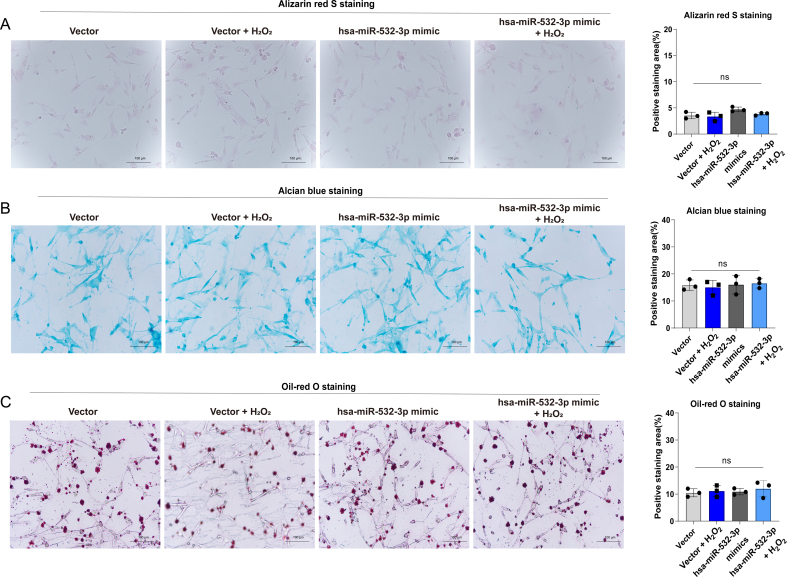
hDMSCs differentiation detection with hsa-miR-532-3p overexpression under H_2_O_2_ intervention. A. Alizarin Red staining for osteogenic differentiation (n = 3); B. Alcian Blue staining for chondrogenic differentiation (n = 3); C. Oil Red O staining for adipogenic differentiation (n = 3). Data represent mean ± SD, ns: no statistical difference.

The hDMSCs extracted early in pregnancy were stained with crystal violet (Fig. 1A). Cell samples from the experimental group were extracted from five patients diagnosed with RSA and five participants who experienced an early miscarriage; their clinical information is listed in the Supplementary Material (Supplementary Table 5). The hDMSCs exhibited an adherent morphology, a spindle shape, equal size, clear edges, and interconnectedness. The viability of hDMSCs was assessed using the CCK8 assay, which revealed robust cell growth after seeding (Fig. 1B). The growth curves of cells at the 1st, 3rd, 5th, 7th, and 9th passages showed similar cellular activities (Supplementary Fig. 1). The flow cytometry analysis revealed negative expression of CD3, CD34, and CD45 in the cells and positive expression of CD29, CD44, CD73, CD90, CD105, and CD146, indicating the successful isolation of hDMSCs in the present study (Fig. 1C–K). EdU and staining experiments were performed to further test the reliability of cell proliferation and differentiation (Supplementary Figs. 2–3).

### 3.2 RSA hDMSCs show a decreased antioxidant capacity

**Fig. 2:**
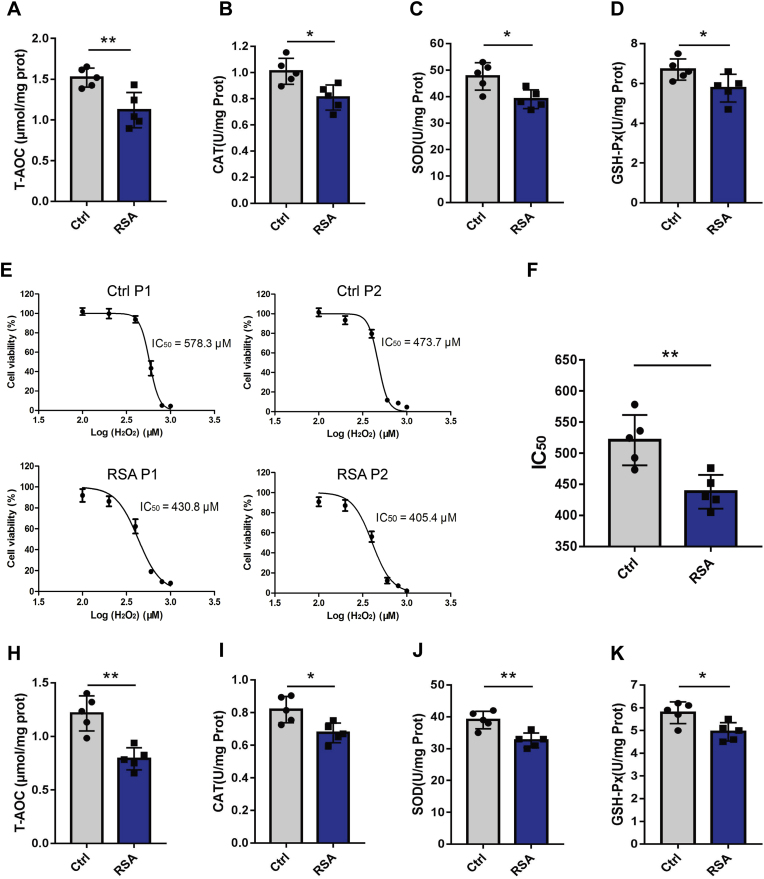
Assessment of antioxidant stress capacity in hDMSCs and decidua tissues. A-D. Comparison of T-AOC, SOD, GSH-PX, and CAT levels in decidua tissues between RSA group (n = 5) and control group (n = 5). E. Two representative cases of the IC_50_ of early pregnancy hDMSCs against H_2_O_2_ from patients with RSA and control group. F. Comparison of the IC_50_ of H_2_O_2_ between the RSA group (n = 5) and control group (n = 5) of hDMSCs. H–K. Comparison of T-AOC, SOD, GSH-PX, and CAT levels in hDMSCs between RSA group (n = 5) and control group (n = 5). Data representing mean ± SD, ∗∗∗*P* < 0.001; ∗∗*P* < 0.01; ∗*P* < 0.05.

The antioxidant capacity of the collected samples, including decidual tissues and hDMSCs collected during early pregnancy, was assessed. Compared with those in the control group, the levels of T-AOC and the enzyme activities of SOD, GSH-PX, and CAT in the decidual tissues of the RSA group were significantly lower (Fig. 2A–D). Additionally, the IC50 of H2O2 for hDMSCs in the RSA group was markedly lower than that in the control group (Fig. 2E–F), indicating increased susceptibility to OS. Furthermore, assessments of the antioxidant capacity revealed significantly lower T-AOC, SOD, GSH-PX, and CAT levels in the hDMSCs of the RSA group than in those of the control group (Fig. 2H–K). These results show that the antioxidant activity of decidual tissue and hDMSCs from RSA patients was lower than that of samples from patients with a normal early pregnancy.

### 3.3 Differential expression of miRNAs in hDMSCs from RSA patients

**Fig. 3:**
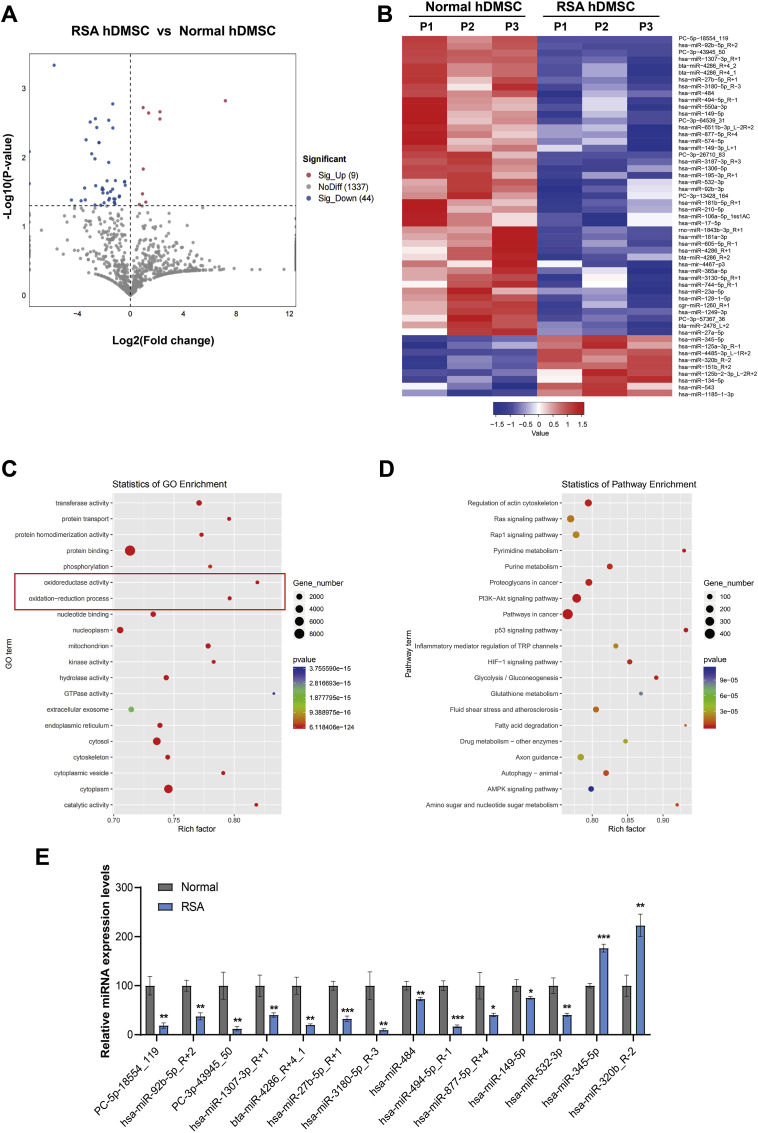
Differential expression of miRNAs and functional enrichment in early pregnancy hDMSCs between patients with RSA group and control group. A. Volcano plot showing the differential expression of miRNAs in both groups (*P* < 0.05). B. Heatmap depicting the differential expression of miRNAs in control group (n = 3) and RSA group (n = 3). C. GO functional enrichment analysis of differentially expressed miRNAs. D. Enrichment analysis of differentially expressed miRNAs through the KEGG signaling pathways. E. Validation of different expression miRNAs in miRNA-sequencing via rt-qPCR. Data representing mean ± SD, ∗∗∗*P* < 0.001; ∗∗*P* < 0.01; ∗*P* < 0.05.

hDMSCs from the RSA and control groups were subjected to miRNA sequencing. Fifty-three differentially expressed miRNAs were identified in hDMSCs between the RSA and control groups, including 44 downregulated and 9 upregulated miRNAs (Supplementary Table 3). All target genes are listed in Supplementary Table 4. A volcano plot and heatmap illustrating the differentially expressed miRNAs are shown in Fig. 3A–B. Furthermore, the GO functional analysis revealed potential targets of the differentially expressed miRNAs that were closely associated with transferase activity, protein transport, protein homodimerization activity, protein binding, phosphorylation, oxidoreductase activity, and oxidation‒reduction processes (Fig. 3C). The KEGG pathway enrichment analysis revealed the enrichment of signaling pathways such as the regulation of the actin cytoskeleton, the Ras signaling pathway, and the Rap1 signaling pathway (Fig. 3D). The results of the GO functional analysis and KEGG pathway enrichment analysis are shown in the Supplementary Material.

### 3.4 Hsa-miR-532-3p antagonizes apoptosis induced by oxidative stress in hDMSCs isolated from patients during early pregnancy

**Fig. 4:**
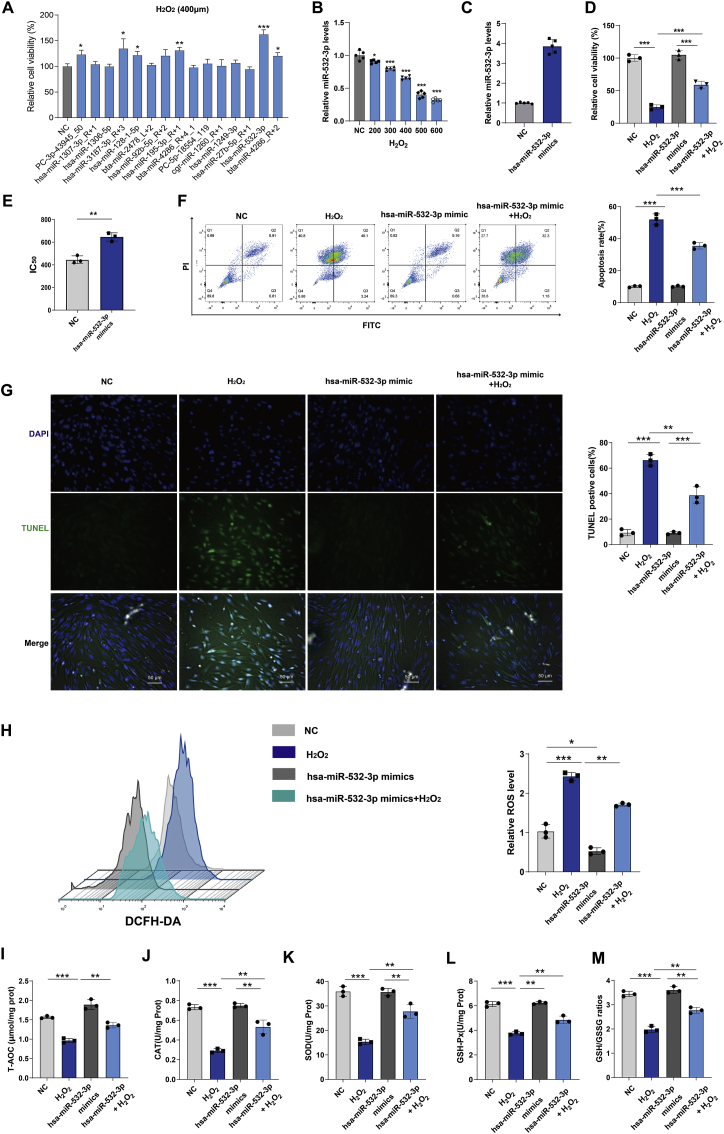
The assessment of hsa-miR-532-3p on anti-oxidative stress in hDMSCs. A. Screening of miRNAs for antagonizing oxidative stress in early pregnancy hDMSCs. The cells were transfected with different miRNA mimics and then subjected to H_2_O_2_ (400 μmol) intervention (n = 3). Cell viability was assessed using CCK8 assay after 12 h of intervention. B. Expression levels of hsa-miR-532-3p in hDMSCs after 12 h of intervention at different concentrations (n = 5). C. Expression levels of hsa-miR-532-3p in hDMSCs transfected with hsa-miR-532-3p mimic (n = 5). D. Changes in cell viability of hDMSCs transfected with hsa-miR-532-3p mimic under H_2_O_2_ intervention (n = 3). E. Comparison of IC_50_ values for H_2_O_2_ intervention in hDMSCs transfected with hsa-miR-532-3p mimic (n = 3). F. Changes in apoptotic cell population of hDMSCs transfected with hsa-miR-532-3p mimic under H_2_O_2_ intervention (n = 3). G. Changes in apoptotic cell population of hDMSCs transfected with hsa-miR-532-3p mimic under H_2_O_2_ intervention (n = 3). H. Measurement of intracellular ROS levels in hDMSCs after different interventions through DCFH-DA probe and flow cytometry (n = 3). I-M. Assessment of T-AOC, CAT, SOD, GSH-PX levels and GSH/GSSG ratios in hDMSCs after the overexpression of hsa-miR-532-3p (n = 3). Data representing mean ± SD, ∗∗∗*P* < 0.001; ∗∗*P* < 0.01; ∗*P* < 0.05.

**Supplementary Fig. 4:**
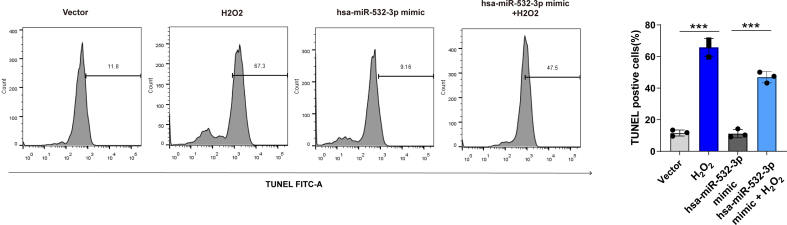
Flow cytometry for TUNEL stain of hDMSCs after different treatment (n = 3). Data represent mean ± SD, ∗∗∗*P* < 0.001.

Among the selected downregulated miRNAs, the top 15 downregulated miRNAs were individually overexpressed using miRNA mimic transfection. hDMSCs were subsequently exposed to H2O2 for 24 h, and cell viability was assessed using the CCK8 assay. As shown in Fig. 4A, after PC-3p-43945_50, hsa-miR-3187-3p_R+3, hsa-miR-128-1-5p, hsa-miR-195-3p, hsa-miR-532-3p, and bta-miR-4286_R+2 were overexpressed, the antioxidant capacity of hDMSCs was significantly increased compared to the control group (P < 0.05). The overexpression of hsa-miR-532-3p resulted in the most significant increase in the antioxidant capacity of hDMSCs. Moreover, the expression of hsa-miR-532-3p gradually decreased in response to increasing concentrations of H2O2 (P < 0.05) (Fig. 4B). The overexpression of hsa-miR-532-3p restored the viability and proliferation of hDMSCs after H2O2 treatment (Fig. 4C–D, Supplementary Fig. 2). Moreover, hsa-miR-532-3p did not affect hDMSC differentiation upon H2O2 treatment (Supplementary Fig. 3). Moreover, the IC50 of H2O2 resistance in hDMSCs was significantly increased after hsa-miR-532-3p overexpression (Fig. 4E), and H2O2-induced hDMSC apoptosis was significantly reduced (Fig. 4F–G, Supplementary Fig. 4). The results revealed that the overexpression of hsa-miR-532-3p led to a decrease in ROS levels in hDMSCs. These findings indicated that hsa-miR-532-3p effectively antagonized the increase in ROS levels induced by H2O2, as illustrated in Fig. 4H. In addition, the overexpression of hsa-miR-532-3p in hDMSCs increased the levels of T-AOC, SOD, GSH-PX, and CAT, indicating increased antioxidant enzyme activity (Fig. 4I–L). The GSH/GSSG ratios were also increased (Fig. 4I–M). In summary, hsa-miR-532-3p antagonized OS-induced apoptosis in hDMSCs isolated during early pregnancy.

### 3.5 Hsa-miR-532-3p regulates NRF2 and GPX4 expression by targeting KEAP1

**Fig. 5:**
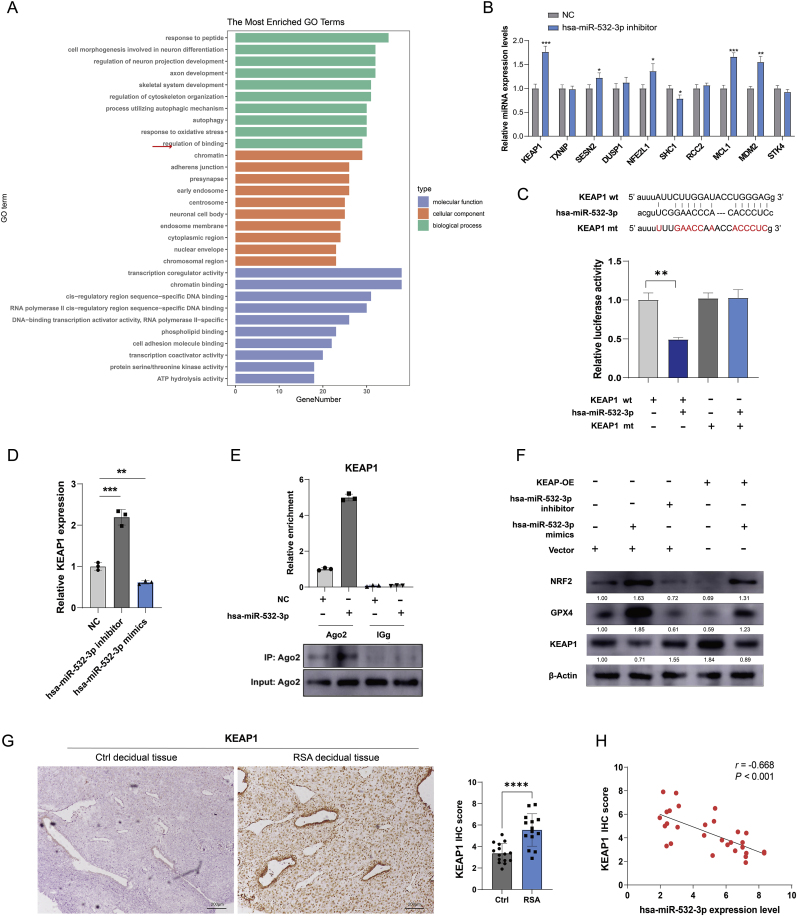
Target interaction of hsa-miR-532-3p with *KEAP1*. A. A total of 703 mRNAs were screened and subjected to GO enrichment analysis. B. The relative miRNA expression changes of top 10 potential target after knockdown of hsa-miR-532-3p (n = 3). C. Bioinformatics prediction of the binding sites between hsa-miR-532-3p and KEAP1, followed by construction of wild-type and mutant luciferase reporter gene plasmids for KEAP1. D. Detection of *KEAP1* expression levels after knockdown or overexpression of hsa-miR-532-3p in hDMSCs (n = 3). E. KEAP1 expression levels in hDMSCs overexpressing hsa-miR-532-3p. F. Western blot analysis of NRF2, GPX4, and KEAP1 expression levels in hDMSCs after knockdown or overexpression of hsa-miR-532-3p. G. Immunohistochemical detection of KEAP1 expression levels in decidua tissues from RSA group (n = 14) and control group (n = 15). H. Analysis of the correlation between hsa-miR-532-3 expression levels and immunohistochemical scoring of KEAP1 in decidua tissues. Data representing mean ± SD, ∗∗*P* < 0.01; ∗∗∗*P* < 0.001; ∗∗∗∗*P* < 0.0001.

**Supplementary Fig. 5:**
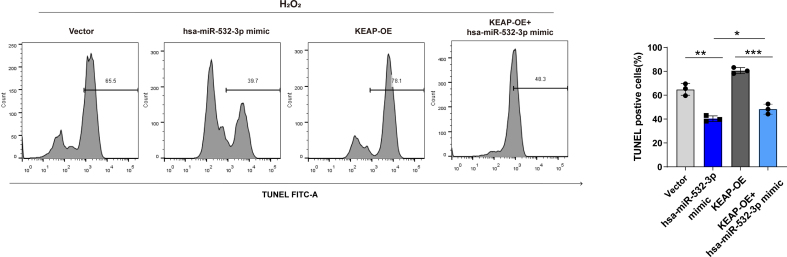
Flow cytometry for TUNEL stain of hDMSCs after overexpression KEAP1 and H_2_O_2_ treatment (n = 3). Data represent mean ± SD, ∗∗∗*P* < 0.001; ∗∗*P* < 0.01; ∗*P* < 0.05.

**Supplementary Fig. 6:**
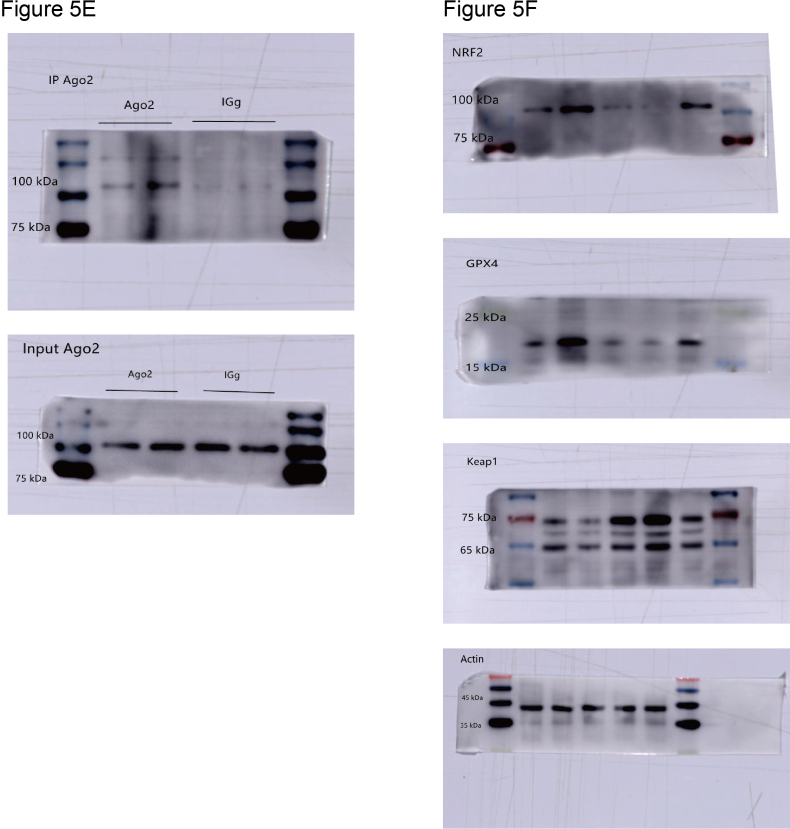
Original picture of western-blot result.

A total of 703 target genes of hsa-miR-532-3p were screened using the miRecords, miRTarBase, and TarBase databases (Supplementary Table 4). For the GO enrichment analysis, we focused on potential targets related to the response to OS (Fig. 5A). As *KEAP1* had the highest relevance score (Supplementary Table 6), we screened for changes in potential target genes after inhibiting hsa-miR-532-3p expression via RT‒qPCR (Fig. 5B). These results revealed that hsa-miR-532-3p responded to OS, possibly via KEAP1. Wild-type and *KEAP1* mutant luciferase reporter genes were constructed to investigate the interaction between *KEAP1* and hsa-miR-532-3p (Fig. 5C). Transfection with the hsa-miR-532-3p mimic significantly inhibited the luciferase activity of the *KEAP1* reporter gene, suggesting a direct interaction between hsa-miR-532-3p and *KEAP1*. Furthermore, KEAP1 overexpression was associated with increased apoptosis in the presence of H2O2 (Supplementary Fig. 5). The knockdown or overexpression of hsa-miR-532-3p in hDMSCs resulted in the upregulation or downregulation of *KEAP1* expression, respectively (Fig. 5D). The overexpression of hsa-miR-532-3p increased the enrichment of *KEAP1* by the Ago2 antibody (Fig. 5E). In addition, hsa-miR-532-3p overexpression suppressed KEAP1 expression and upregulated NRF2 and GPX4 expression, whereas hsa-miR-532-3p knockdown promoted KEAP1 expression and downregulated NRF2 and GPX4 expression. Compared with the increased expression caused by hsa-miR-532-3p alone, co-overexpression of hsa-miR-532-3p and KEAP1 counteracted the upregulation of NRF2 and GPX4 (Fig. 5F). The immunohistochemical analysis of the samples from the RSA group revealed significantly higher expression of KEAP1 than in those from the control group (P < 0.05) (Fig. 5G). Moreover, the expression level of hsa-miR-532-3p was negatively correlated with the immunohistochemical score for *KEAP1* (r = −0.668, P < 0.05) (Fig. 5H). These results indicate that hsa-miR-532-3p targets *KEAP1* to regulate NRF2 and GPX4 expression. The original Western blot results are shown in Supplementary Fig. 6.

### 3.6 Hsa-miR-532-3p antagonizes the decrease of antioxidant stress via KEAP1 in hDMSCs

**Fig. 6:**
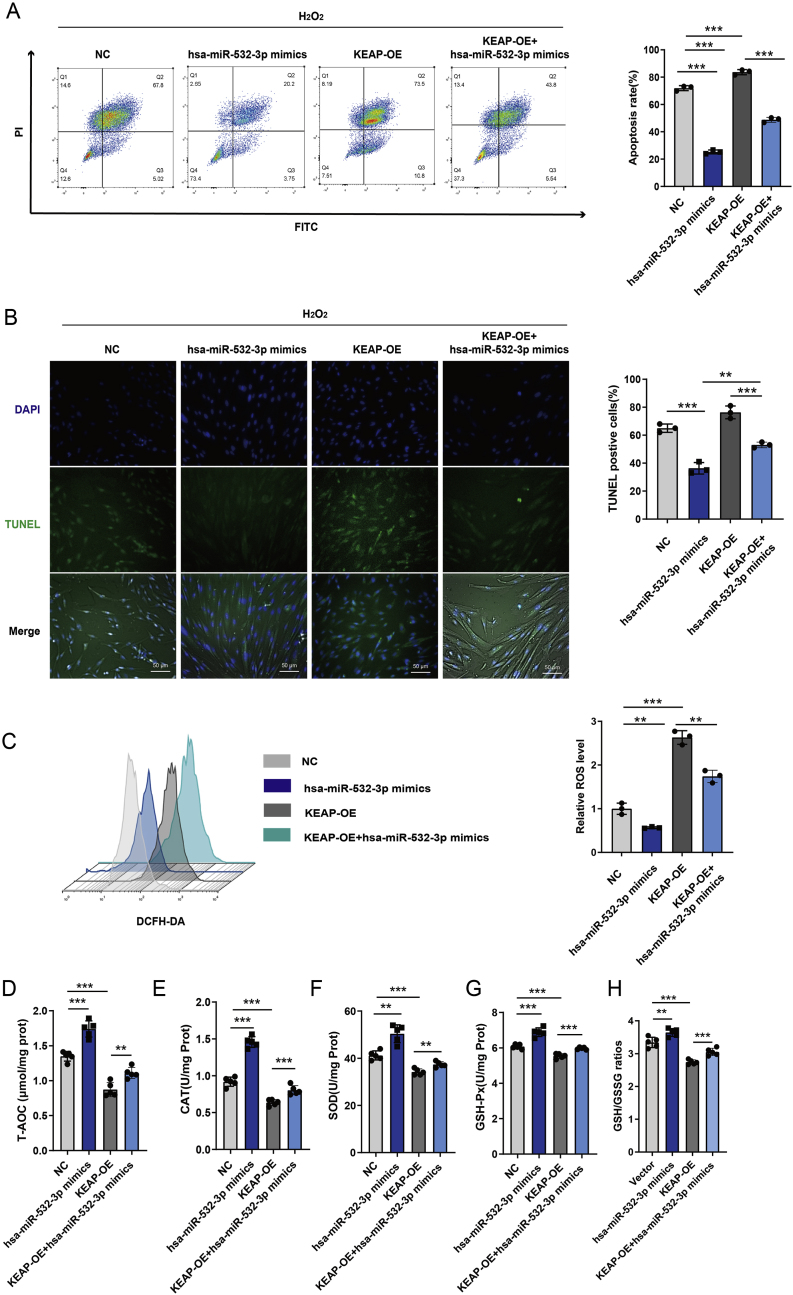
Hsa-miR-532-3p antagonizes the decrease of antioxidant capacity via KEAP1 in hDMSCs. A. Flow cytometer was used to detect the changes in cell apoptosis in hDMSCs after transfection with hsa-miR-532-3p mimic and KEAP1 following H_2_O_2_ intervention (n = 3). B. TUNEL staining was performed to detect the changes in cell apoptosis in hDMSCs after transfection with hsa-miR-532-3p mimic and KEAP1 following H_2_O_2_ intervention (n = 3). C. DCFH-DA probe and flow cytometer were used to detect the levels of ROS in hDMSCs after different interventions (n = 3). D-H. The total antioxidant capacity (T-AOC), catalase (CAT), superoxide dismutase (SOD), and glutathione peroxidase (GSH-PX) levels and GSH/GSSG ratios were compared in hDMSCs after transfection with hsa-miR-532-3p mimic and KEAP1 (n = 5). Data representing mean ± SD, ∗∗∗*P* < 0.001; ∗∗*P* < 0.01; ∗*P* < 0.05.

Upon H2O2 stimulation and the simultaneous overexpression of hsa-miR-532-3p and KEAP1 in hDMSCs, hsa-miR-532-3p antagonized KEAP1-mediated apoptosis (Fig. 6A–B). H2O2-induced OS decreased the antioxidant capacity of hDMSCs isolated during early pregnancy. However, KEAP1 overexpression in hDMSCs significantly decreased the T-AOC, the activities of SOD, GSH-PX, and CAT, and the GSH/GSSH ratios. In contrast, hsa-miR-532-3p reversed the decreases in T-AOC levels, the enzymatic activities of CAT, SOD, and GSH-PX, and the GSH/GSSH ratio induced by H2O2 in hDMSCs overexpressing KEAP1 (Fig. 6C–F). These results indicate that hsa-miR-532-3p can counteract the weakened antioxidant response mediated by KEAP1.

### 3.7 Upregulation of hsa-miR-532-3p expression inhibits KEAP1 expression in decidual tissue and reduces mouse embryo miscarriage

**Fig. 7:**
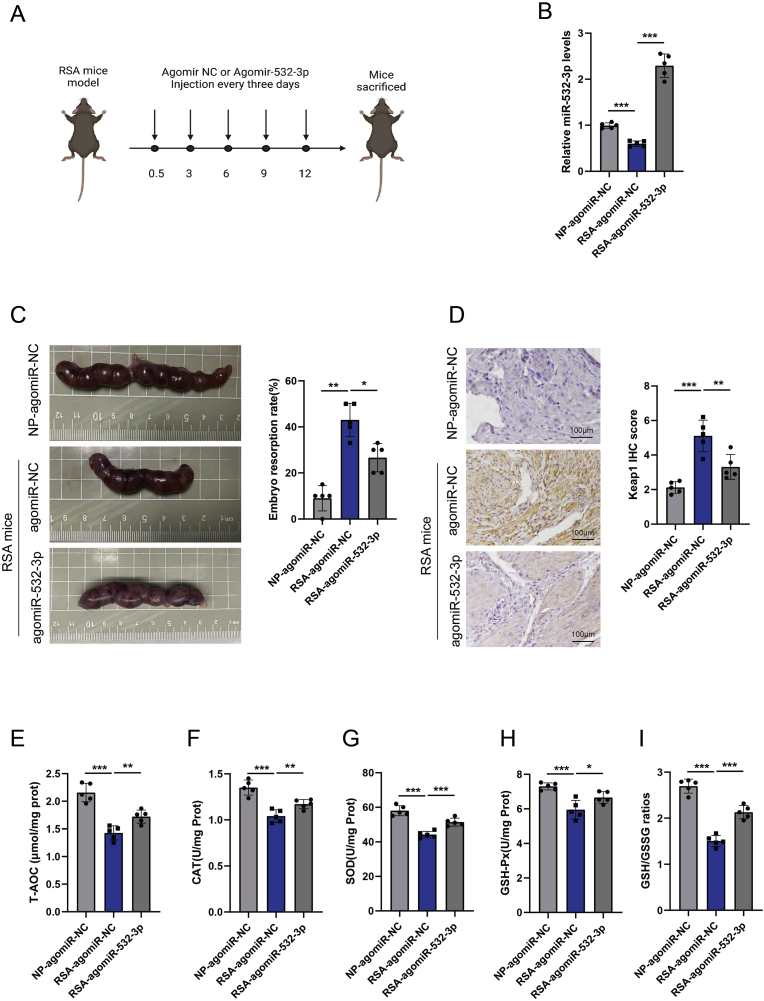
The inhibitory effect of hsa-miR-532-3p on RSA in mice. A. Construction of the RSA model and tail vein injection method of agomir-532-3p. B. Expression levels of miR-532-3p in decidual tissues of different groups (n = 5). C. Comparison of embryo conditions and absorption rates in different groups (n = 5). D. Comparison of KEAP1 expression levels in decidual tissues of different groups (n = 5). E-I. Comparison of T-AOC, SOD, GSH-PX, CAT levels and GSH/GSSG ratios in different groups (n = 5). Data representing mean ± SD, ∗∗∗*P* < 0.001, ∗∗*P* < 0.01, ∗*P* < 0.05.

A mouse model of RSA was established, and agomir-532-3p was intravenously injected via the tail vein (Fig. 7A). The expression of miR-532-3p was decreased in the decidual tissues of the RSA group compared with those of the normal pregnancy group treated with agomiR-NC (NP-agomiR-NC), whereas it was significantly upregulated in the RSA-agomir-532-3p group (RSA group treated with agomir-532-3p) (Fig. 7B). The overexpression of miR-532-3p reduced the absorption rate of embryos in the RSA group (Fig. 7C) and significantly downregulated KEAP1 expression (Fig. 7D). Furthermore, compared with RSA mice treated with agomiR-NC, RSA mice treated with miR-532-3p presented significantly higher T-AOC levels, enzymatic activities of SOD, GSH-PX, and CAT, and GSH/GSSG ratios (Fig. 7E–I). These results revealed that the expression of hsa-miR-532-3p can inhibit KEAP1 expression in decidual tissue and reduce mouse embryo miscarriage.

### 3.8 Correlation analysis between serum hsa-miR-532-3p levels and clinical characteristics

**Fig. 8:**
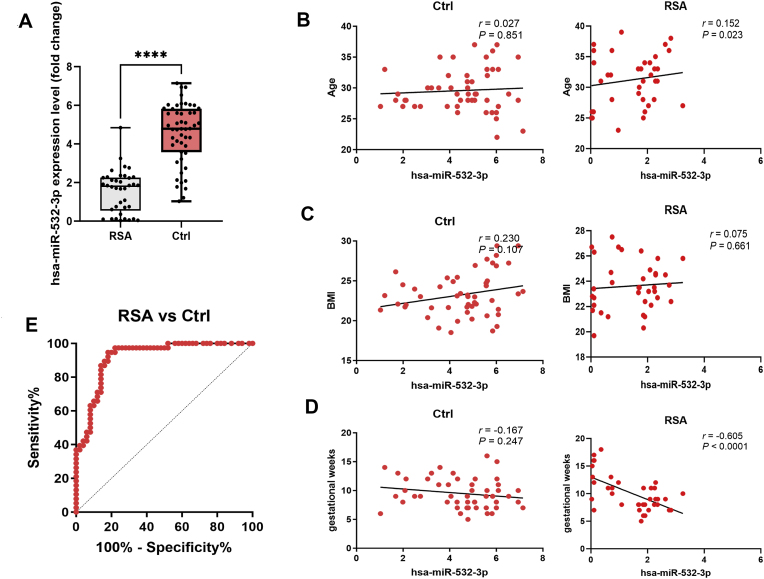
Hsa-miR-532-3p is potential biomarker for RSA.A. Comparison of serum hsa-miR-532-3p levels between control group (n = 50) and RSA group (n = 38), ∗∗∗∗, *P* < 0.0001. B. Correlation analysis between serum hsa-miR-532-3p levels and age, control group (n = 50), RSA group (n = 38). C. Correlation analysis between serum hsa-miR-532-3p levels and BMI, control group (n = 50), RSA group (n = 38). D. Correlation analysis between serum hsa-miR-532-3p levels and gestational week, control group (n = 50), RSA group (n = 38). E. Diagnostic value of serum hsa-miR-532-3p expression in RSA patients assessed by ROC curve.

Finally, we evaluated the correlations between serum hsa-miR-532-3p levels and the clinical characteristics of patients with RSA. Thirty-eight patients with RSA were included in the experimental group, and fifty women with primary miscarriages during early pregnancy (7th-11th weeks of pregnancy) were included in the control group. The basic clinical characteristics of the patients are shown in Table 1. A comparison of the serum levels of hsa-miR-532-3p between the RSA group and the control group revealed significantly lower serum levels of hsa-miR-532-3p in the RSA group than those in the control group (P < 0.05) (Fig. 8A). An analysis of the correlations between the serum hsa-miR-532-3p level and the clinical characteristics of the two groups of pregnant women revealed no significant correlations between the serum hsa-miR-532-3p level and BMI or gestational age (P > 0.05) (Fig. 8B–C). A strong negative correlation was observed with gestational age in the RSA group (r = −0.479, P = 0.002), but this correlation was not statistically significant in the control group (P > 0.05), as shown in Fig. 8D. The AUC of serum hsa-miR-532-3p levels for predicting the RSA diagnosis in patients with primary recurrent miscarriage was 0.9184, with a sensitivity of 94.746 % and an estimated specificity of 82.0 %. The diagnostic cutoff point was 2.941 (Fig. 8E). These findings indicate that the serum hsa-miR-532-3p level has good predictive value for identifying RSA.

**Table 1:** basic characteristics of patients.

	Control group(n = 50)	RSA group(n = 38)	t	P
Age	29.58 ± 3.447	31.16 ± 4.175	t = 1.941	0.055
BMI(kg/m^2^)	23.27 ± 2.70	23.65 ± 1.91	t = 0.735	0.464
Gestational week	9.50 ± 2.573	9.97 ± 3.24	t = 0.030	0.863
Smoking history(%)	3(6.0)	2(5.2)	–	>0.999
Alcohol history(%)	4(8.0)	2(5.2)	–	0.695

## 4 Discussion

HDMSCs are involved in the generation, maintenance, repair, and formation of branching villous structures in placental tissue, which are indispensable for maintaining placental function and normal fetal development during early pregnancy and are closely related to the occurrence of RSA [42]. miRNAs, small noncoding RNAs, regulate gene expression by binding to the 3′UTRs of target genes in a sequence-specific manner, thereby reducing gene expression, and are pivotal in numerous biological processes, such as cell growth, differentiation, metabolism, immunity, cancer, and autoimmune disorders [43]. Several studies have suggested potential associations between miRNAs and different types of RSA [31,44]. However, studies examining whether miRNAs interact with hDMSCs and their underlying relationships are still lacking. The present study identified hsa-miR-532-3p as one of the differentially expressed miRNAs in patients with RSA compared with women with miscarriages during early pregnancy. Its overexpression mitigates the adverse effects of OS on hDMSCs. Importantly, we provide strong evidence that hsa-miR-532-3p protects hDMSCs and reduces OS-induced damage by targeting the KEAP1/NRF2 pathway in hDMSCs. Furthermore, the serum hsa-miR-532-3p level in patients with RSA was negatively correlated with gestational age in the clinic. Therefore, hsa-miR-532-3p may serve as a promising predictive biomarker of RSA.

Accumulating evidence has shown a potential relationship between OS and RSA [24,45]. ROS are strongly associated with female reproduction and fertility processes and act as important mediators of steroidogenesis of the ovary, hormone signaling, ovulation, corpus luteum formation, oocyte maturation, luteal maintenance during pregnancy, implantation, compaction, blastocyst development, and germ cell function [26]. During pregnancy, the placenta is in an active metabolic state and continuously generates ROS [46]. Maintaining a precise equilibrium between the production and accumulation of ROS and the ability of the biological system to detoxify these reactive products are crucial for normal pregnancy. An imbalance in the redox state can precipitate adverse outcomes such as spontaneous abortion, preeclampsia, or intrauterine growth restriction [47]. If OS occurs too soon during pregnancy, it can impair placental growth and exacerbate syncytiotrophoblast degeneration, leading to miscarriage [24]. Moreover, elevated levels of HSP70, nitrotyrosine, and apoptotic markers in the villi of placentas from individuals who experienced an abortion indicate oxidative damage, ultimately leading to pregnancy termination [19,48]. In this study, we explored the antioxidant capacity of decidual tissues and hDMSCs by assessing T-AOC, SOD, GSH-PX, and CAT levels. The antioxidant capacity of both decidual tissues and hDMSCs in patients was significantly lower than that in women with abortion during early pregnancy, which confirmed the possible relationship between RSA and the antioxidant capacity. Furthermore, since H2O2 is one of the most prevalent ROS, the IC50 value determined after the H2O2 intervention in patients with RSA was significantly lower than that in women who experienced a first miscarriage during early pregnancy. Because hDMSCs play a crucial role in promoting decidual tissue development in early pregnancy, the excessive ROS generated by placental trophoblast tissue during early gestational weeks can lead to OS [49]. If the capability of antioxidants to address OS is diminished, hDMSCs become more vulnerable to the detrimental effects of ROS, resulting in reduced cell viability, apoptosis, and impaired decidual formation. This finding may represent a significant factor contributing to the pathophysiology of RSA, highlighting the potential role of oxidative stress in the occurrence and progression of this condition. Additionally, we selected key differentially expressed miRNAs from the downregulated miRNAs in hDMSCs from the RSA group to validate the prevention of OS-induced damage.

Previous studies have reported the differential expression of miRNAs in patients with RSA, such as the upregulation of hsa-miR-184, hsa-miR-187, and hsa-miR-125b-2, as well as the downregulation of hsa-miR-520f, hsa-miR-3175, and hsa-miR-4672 [33]. Furthermore, the regulation of miRNAs is intricately associated with OS and plays a pivotal role in various pathological processes and diseases, including cancer, ischemic stroke, and vascular disorders [[50], [51], [52]]. However, a comprehensive exploration of the differential expression of miRNAs in hDMSCs is still lacking. This study is the first to identify 53 differentially expressed miRNAs in the hDMSCs of patients with RSA and women with a first miscarriage. Interestingly, the overexpression of PC-3p-43945_50, hsa-miR-3187-3p_R+3, hsa-miR-128-1-5p, hsa-miR-195-3p, hsa-miR-532-3p, and bta-miR-4286_R+2 significantly increased the antioxidant capacity of hDMSCs. The overexpression of hsa-miR-532-3p resulted in the greatest change in the antioxidant capacity. The function and mechanism of miR-532-3p remain incompletely understood, although it has been extensively studied in the context of various cancers, including colorectal cancer [53,54], liver cancer [55], lung cancer [56] and renal cancer [57]. It plays a critical role in regulating cellular processes and is differentially expressed in different cancers, where it acts as a tumor suppressor by inhibiting proliferation [56,58,59]. For example, it reduces the metastatic and proliferative abilities of lung cancer cells by modulating FOXP3 levels and negatively regulating Rab3IP levels to suppress gastric cancer cell proliferation [56,60]. Moreover, miR-532-3p has also been reported to act as a crucial regulator of OS in ischemic stroke, targeting NADPH oxidase 2, which is responsible for the generation of ROS in brain tissues [52]. Our study revealed the significant downregulation of hsa-miR-532-3p expression in response to H2O2 exposure. Interestingly, after hDMSCs were transfected with hsa-miR-532-3p mimics, their resistance to H2O2 increased significantly, with relatively high levels of T-AOC, SOD, GSH-PX, and CAT. These findings clearly demonstrate the improved antioxidant capacity of hDMSCs overexpressing hsa-miR-532-3p. Thus, the expression of hsa-miR-532-3p was positively associated with the protection of hDMSCs against H2O2-induced apoptosis and OS-mediated damage.

The present study also revealed the potential targets and functional enrichment of hsa-miR-532-3p, as well as its important role in OS. Through a database analysis, we identified possible interactions between hsa-miR-532-3p and *KEAP1*. To the best of our knowledge, this study is the first to elucidate the regulatory mechanism by which hsa-miR-532-3p targets *KEAP1* in hDMSCs from patients with RSA. The human body possesses a highly efficient defense system that serves to detoxify and eliminate harmful chemicals while also deactivating ROS to counteract the damage caused by excessive levels of ROS. NRF2 functions as a central regulator of cellular responses to environmental stress by inducing the expression of detoxification and antioxidant enzymes [28]. It is the main regulator of multiple antioxidant enzymes and maintains the cellular redox balance by stimulating the activity of antioxidant defense components such as SOD, GSH-Px, heme oxygenase-1 (HO-1), glutathione reductase, thioredoxin reductase, and ferritin [61,62]. KEAP1, discovered in 1999, is the main negative regulator of NRF2 and possesses a broad and complex track-like structure at its N-terminus [63]. *KEAP1* serves as a target site for Cullin-dependent E3 ubiquitin ligases and degrades NRF2 through ubiquitination [64,65]. Most NRF2 inducers, such as tert-butylhydroquinone (tBHQ) and oleanolic acid, are electrophilic and readily react with the cysteine residues of KEAP1, activating the KEAP1-NRF2 signaling pathway and triggering protective antioxidant responses [66,67]. The KEAP1-NRF2 pathway plays a critical role in regulating cellular sensitivity to chemical and oxidative stress by controlling the basal and inducible expression of detoxification and antioxidant enzymes, as well as protecting vital detoxification organs [68,69]. Moreover, this signaling pathway is strongly related to many life activities, including redox signaling and homeostasis, drug metabolism and disposition, intermediary metabolism, the cellular adaptation to stress, chemoprevention and chemoresistance, toxicity, inflammation, neurodegeneration, lipogenesis, and aging [70]. In the context of OS, KEAP1 undergoes conformational changes leading to the dissociation of NRF2, which upregulates NRF2 expression, allowing it to accumulate in the cell nucleus and form heterodimers with muscle aponeurotic fibrosarcoma (Maf) [67,71]. These heterodimers bind to antioxidant response elements (AREs), initiating the transcription of various antioxidants and reducing OS-induced damage [72,73]. In this study, we confirmed that *KEAP1* is a potential target of hsa-miR-532-3p through GO functional analysis and KEGG pathway enrichment analysis. Under OS conditions, hsa-miR-532-3p expression is downregulated, resulting in decreased targeting of *KEAP1*. This process results in elevated KEAP1 protein levels, which subsequently lead to the downregulation of NRF2 expression, further impairing the expression of key antioxidant factors such as GPX4. Consequently, the antioxidant capacity is diminished. The KEAP1–NRF2 pathway regulates a network of highly inducible proteins that protect aerobic cells from the damaging effects of reactive oxygen species and toxic electrophiles, which are key contributors to neoplastic and chronic degenerative diseases [74]. When activated, KEAP1–NRF2 promotes survival and antioxidant activity, while its cross-talk with other signaling pathways influences essential processes such as cell proliferation, apoptosis, angiogenesis, and metastasis [75]. The KEAP1–NRF2 signaling pathway, along with AREs, serves as a critical defense mechanism against OS [76]. Moreover, the concurrent overexpression of hsa-miR-532-3p and KEAP1 in hDMSCs revealed that hsa-miR-532-3p has an antagonistic effect on KEAP1-mediated apoptosis. Thus, our study elucidated the possible mechanism by which hsa-miR-532-3p regulates the reduction in OS-induced damage in hDMSCs, highlighting hsa-miR-532-3p as a critical target for protecting hDMSCs from OS-induced damage.

We performed a validation assay using blood samples from both the RSA and control groups to further explore the clinical value of our findings. The results revealed that hsa-miR-532-3p was reliably detected in the peripheral sera from both groups. Interestingly, in the RSA group, the serum hsa-miR-532-3p level was strongly negatively correlated with the gestational age, but this correlation was not statistically significant in the control group. The ROC curve also revealed the critical predictive value of serum hsa-miR-532-3p levels as a biomarker for the early diagnosis of RSA. These findings suggest that hsa-miR-532-3p may have a protective effect on decidual tissue and embryonic development in patients with RSA and could serve as an effective target for the prevention and treatment of RSA. However, this study has several limitations. This study included a relatively small number of subjects and lacked additional clinical data for analysis. We exclusively investigated the miRNAs exhibiting the greatest alterations; however, other differentially expressed miRNAs warrant further investigation. Therefore, further clinical studies with larger sample sizes are warranted.

## 5 Conclusions

This study identified differentially expressed miRNAs in the hDMSCs of RSA early pregnancy patients and identified hsa-miR-532-3p with anti-oxidative stress response capabilities. We found that hsa-miR-532-3p protected hDMSCs from OS-induced damage by targeting the KEAP1/NRF2 pathway, thereby reducing the occurrence of RSA. Therefore, hsa-miR-532-3p is a potential biomarker for the diagnosis and treatment of RSA.

## CRediT authorship contribution statement

**Hong Zhou:** Formal analysis, Data curation, Conceptualization. **Jiaxin Zhou:** Writing – review & editing, Writing – original draft, Methodology, Investigation, Formal analysis, Data curation. **ShanShan Liu:** Validation, Software, Resources, Data curation. **Jing Niu:** Software, Resources, Project administration, Methodology, Investigation. **Jinghua Pan:** Writing – review & editing, Resources, Project administration, Investigation, Funding acquisition. **Ruiman Li:** Writing – review & editing, Writing – original draft, Validation, Supervision, Project administration, Conceptualization.

## Funding statement

This work was partially supported by the Guangzhou science and technology plan project (2025A04J3471), Guangdong Province Medical Science and Technology Research Fund Project (A2021056), 10.13039/501100012245Science and Technology Planning Project of Guangdong Province of China (No.2022A1515012139), and Clinical Frontier Technology Program of the First Affiliated Hospital of 10.13039/501100004024Jinan University, China (No. JNU1AF-CFTP-2022-a01209).

## Declaration of competing interest

The authors declare that the research was conducted in the absence of any commercial or financial relationships that could be construed as a potential conflict of interest.

## Data Availability

Data will be made available on request.

## Appendix A Supplementary data

Supplementary Table 1 The sequences of miRNA mimics. Supplementary Table 1

Supplementary Table 2 The sequences of rt-qPCR. Supplementary Table 2

Supplementary Table 3 Differential expression of miRNAs in RSA group compared with control group (TOP 50). Supplementary Table 3

Supplementary Table 4 Target gene list. Supplementary Table 4

Supplementary Table 5 Information of sample providers. Supplementary Table 5

Supplementary Table 6 The hsa-miR-532-3p potential targets for response to oxidative stress. (see supplementary data). Supplementary Table 6

Supplementary material 1 GO functional analysis. Supplementary material 1

Supplementary material 2 KEGG pathway enrichment analysis. Supplementary material 2
