# Supplementary material for: Hsa-miR-532-3p protects human decidual mesenchymal stem cells from oxidative stress in recurrent spontaneous abortion via targeting KEAP1
Source: Redox Biol. 2025 Feb 1;80:103508. doi: 10.1016/j.redox.2025.103508 (PMC11847473; doi:10.1016/j.redox.2025.103508)
Supplement: Supplementary Table 1 — The sequences of miRNA mimics. [file mmc1.docx]

Supplementary Table 1: The sequences of miRNA mimics

| miRNA name | miRNA seqence |
| --- | --- |
| PC-3p-43945_50 | TCCCTCTCCCTCCTTGCTCCTAC |
| hsa-miR-1307-3p_R+1 | ACTCGGCGTGGCGTCGGTCGTGG |
| hsa-miR-1306-5p | CCACCTCCCCTGCAAACGTCCA |
| hsa-miR-3187-3p_R+3 | TTGGCCATGGGGCTGCGCGGGGC |
| hsa-miR-128-1-5p | CGGGGCCGTAGCACTGTCTGAGA |
| bta-miR-2478_L+2 | TCGTATCCCACTTCTGACACCA |
| hsa-miR-92b-5p_R+2 | AGGGACGGGACGCGGTGCAGTGTT |
| hsa-miR-195-3p_R+1 | CCAATATTGGCTGTGCTGCTCCA |
| bta-miR-4286_R+4_1 | ACCCCACTCCTGGTACCAAAA |
| PC-5p-18554_119 | TTCGGAGATAGGGCTCAGC |
| cgr-miR-1260_R+1 | ATCCCACCGCTGCCACCAG |
| hsa-miR-1249-3p | ACGCCCTTCCCCCCCTTCTTCA |
| hsa-miR-27b-5p_R+1 | AGAGCTTAGCTGATTGGTGAACA |
| hsa-miR-532-3p | CCTCCCACACCCAAGGCTTGCA |
| bta-miR-4286_R+2 | ACCCCACTCCTGGTACCAA |
