# Supplementary material for: Hsa-miR-532-3p protects human decidual mesenchymal stem cells from oxidative stress in recurrent spontaneous abortion via targeting KEAP1
Source: Redox Biol. 2025 Feb 1;80:103508. doi: 10.1016/j.redox.2025.103508 (PMC11847473; doi:10.1016/j.redox.2025.103508)
Supplement: Supplementary Table 2 — The sequences of rt-qPCR. [file mmc2.docx]

Supplementary Table 2: The sequences of rt-qPCR

| Target |  | miRNA seqence |
| --- | --- | --- |
| hsa-miR-532-3p | Forward | 5'-GCGCCCCCACACCCAA-3' |
|  | Reverse | 5'-AGTGCAGGGTCCGAGGTATT-3' |
| PC-5p-18554_119 | Forward | 5'-GCGCGTTCGGAGATAGGG-3' |
|  | Reverse | 5'-AGTGCAGGGTCCGAGGTATT-3' |
| hsa-miR-92b-5p_R+2 | Forward | 5'-GGACGGGACGCGGTGC-3' |
|  | Reverse | 5'-AGTGCAGGGTCCGAGGTATT-3' |
| PC-3p-43945_50 | Forward | 5'-CGTCCCTCTCCCTCCTTGC-3' |
|  | Reverse | 5'-AGTGCAGGGTCCGAGGTATT-3' |
| hsa-miR-1307-3p_R+1 | Forward | 5'-CTCGGCGTGGCGTCGG-3' |
|  | Reverse | 5'-AGTGCAGGGTCCGAGGTATT-3' |
| bta-miR-4286_R+4_1 | Forward | 5'-GCGACCCCACTCCTGGTA-3' |
|  | Reverse | 5'-AGTGCAGGGTCCGAGGTATT-3' |
| hsa-miR-27b-5p_R+1 | Forward | 5'-CGCGAGAGCTTAGCTGATTGG-3' |
|  | Reverse | 5'-AGTGCAGGGTCCGAGGTATT-3' |
| hsa-miR-3180-5p_R-3 | Forward | 5'-GCTTCCAGACGCTCCGC-3' |
|  | Reverse | 5'-AGTGCAGGGTCCGAGGTATT-3' |
| hsa-miR-484 | Forward | 5'-GCGTCAGGCTCAGTCCCCT-3' |
|  | Reverse | 5'-AGTGCAGGGTCCGAGGTATT-3' |
| hsa-miR-494-5p_R-1 | Forward | 5'-GCGAGGTTGTCCGTGTTGT-3' |
|  | Reverse | 5'-AGTGCAGGGTCCGAGGTATT-3' |
| hsa-miR-877-5p_R+4 | Forward | 5'-CGGTAGAGGAGATGGCGCAG-3' |
|  | Reverse | 5'-AGTGCAGGGTCCGAGGTATT-3' |
| hsa-miR-149-5p | Forward | 5'-CGTCTGGCTCCGTGTCTTC-3' |
|  | Reverse | 5'-AGTGCAGGGTCCGAGGTATT-3' |
| hsa-miR-345-5p | Forward | 5'-GCGGCTGACTCCTAGTCCA-3' |
|  | Reverse | 5'-AGTGCAGGGTCCGAGGTATT-3' |
| hsa-miR-320b_R-2 | Forward | 5'-CGCGAAAAGCTGGGTTGA-3' |
|  | Reverse | 5'-AGTGCAGGGTCCGAGGTATT-3' |
| U6 | Forward | 5'-CTCGCTTCGGCAGCACA-3' |
|  | Reverse | 5'-AACGCTTCACGAATTTGCGT-3' |
| KEAP1 | Forward | 5'-CTGGAGGATCATACCAAGCAGG-3' |
|  | Reverse | 5'-GGATACCCTCAATGGACACCAC-3' |
| GAPDH | Forward | 5'-GATTCCACCCATGGCAAATTC-3' |
|  | Reverse | 5'-CTGGAAGATGGTGATGGGATT-3' |
