# Supplementary material for: Hsa-miR-532-3p protects human decidual mesenchymal stem cells from oxidative stress in recurrent spontaneous abortion via targeting KEAP1
Source: Redox Biol. 2025 Feb 1;80:103508. doi: 10.1016/j.redox.2025.103508 (PMC11847473; doi:10.1016/j.redox.2025.103508)
Supplement: Supplementary Table 3 — Differential expression of miRNAs in RSA group compared with control group (TOP 50). [file mmc3.docx]

Supplemental Table 3. Differential expression of miRNAs in RSA group compared with control group (TOP 50)

| Index | miRNA_name | up/down | Fold_change  (RSA/Ctrl) | log2  (fold_change) | P  (t_test) |
| --- | --- | --- | --- | --- | --- |
| 1 | PC-3p-43945_50 | down | 0.02 | -5.76 | 4.60E-04 |
| 2 | hsa-miR-4485-3p_L-1R+2 | up | 143.26 | 7.16 | 1.51E-03 |
| 3 | hsa-miR-1307-3p_R+1 | down | 0.40 | -1.33 | 1.68E-03 |
| 4 | hsa-miR-345-5p | up | 1.94 | 0.96 | 1.90E-03 |
| 5 | hsa-miR-151b_R+2 | up | 4.68 | 2.23 | 2.18E-03 |
| 6 | hsa-miR-320b_R-2 | up | 2.59 | 1.37 | 2.27E-03 |
| 7 | hsa-miR-1306-5p | down | 0.16 | -2.65 | 2.75E-03 |
| 8 | hsa-miR-1185-1-3p | up | 4.67 | 2.22 | 2.77E-03 |
| 9 | hsa-miR-3187-3p_R+3 | down | 0.32 | -1.63 | 2.89E-03 |
| 10 | hsa-miR-128-1-5p | down | 0.12 | -3.04 | 3.07E-03 |
| 11 | bta-miR-2478_L+2 | down | 0.17 | -2.54 | 3.69E-03 |
| 12 | hsa-miR-92b-5p_R+2 | down | 0.40 | -1.32 | 3.75E-03 |
| 13 | hsa-miR-195-3p_R+1 | down | 0.10 | -3.35 | 5.56E-03 |
| 14 | bta-miR-4286_R+4_1 | down | 0.19 | -2.37 | 6.11E-03 |
| 15 | bta-miR-4286_R+4_2 | down | 0.19 | -2.37 | 6.11E-03 |
| 16 | PC-5p-18554_119 | down | -inf | -inf | 8.23E-03 |
| 17 | cgr-miR-1260_R+1 | down | 0.13 | -2.92 | 8.87E-03 |
| 18 | hsa-miR-1249-3p | down | 0.16 | -2.68 | 1.05E-02 |
| 19 | hsa-miR-27b-5p_R+1 | down | 0.32 | -1.63 | 1.18E-02 |
| 20 | hsa-miR-125a-3p_R-1 | up | 1.95 | 0.97 | 1.48E-02 |
| 21 | hsa-miR-532-3p | down | 0.46 | -1.13 | 2.17E-02 |
| 22 | bta-miR-4286_R+2 | down | 0.30 | -1.76 | 2.19E-02 |
| 23 | hsa-miR-484 | down | 0.74 | -0.43 | 2.24E-02 |
| 24 | hsa-miR-23a-5p | down | 0.73 | -0.45 | 2.26E-02 |
| 25 | PC-3p-26710_83 | down | -inf | -inf | 2.40E-02 |
| 26 | hsa-miR-149-5p | down | 0.76 | -0.39 | 2.53E-02 |
| 27 | hsa-miR-494-5p_R-1 | down | 0.16 | -2.62 | 2.57E-02 |
| 28 | hsa-miR-210-5p | down | 0.23 | -2.12 | 2.77E-02 |
| 29 | hsa-miR-3180-5p_R-3 | down | 0.09 | -3.46 | 2.78E-02 |
| 30 | hsa-miR-877-5p_R+4 | down | 0.39 | -1.34 | 2.89E-02 |
| 31 | PC-3p-13428_164 | down | 0.23 | -2.14 | 2.91E-02 |
| 32 | rno-miR-1843b-3p_R+1 | down | 0.24 | -2.07 | 2.92E-02 |
| 33 | hsa-miR-181a-3p | down | 0.35 | -1.50 | 3.12E-02 |
| 34 | hsa-miR-550a-3p | down | 0.24 | -2.08 | 3.28E-02 |
| 35 | hsa-miR-149-3p_L+1 | down | 0.30 | -1.73 | 3.29E-02 |
| 36 | hsa-miR-134-5p | up | 1.88 | 0.91 | 3.38E-02 |
| 37 | hsa-miR-92b-3p | down | 0.56 | -0.83 | 3.64E-02 |
| 38 | hsa-miR-181b-5p_R+1 | down | 0.56 | -0.85 | 3.73E-02 |
| 39 | hsa-miR-574-5p | down | 0.33 | -1.58 | 3.93E-02 |
| 40 | hsa-miR-27a-5p | down | 0.42 | -1.25 | 3.99E-02 |
| 41 | hsa-miR-6511b-3p_L-2R+2 | down | 0.45 | -1.16 | 4.02E-02 |
| 42 | hsa-miR-605-5p_R-1 | down | 0.09 | -3.41 | 4.13E-02 |
| 43 | hsa-miR-365a-5p | down | 0.32 | -1.63 | 4.15E-02 |
| 44 | PC-3p-57367_36 | down | 0.05 | -4.45 | 4.15E-02 |
| 45 | PC-3p-64539_31 | down | 0.08 | -3.73 | 4.26E-02 |
| 46 | hsa-miR-125b-2-3p_L-2R+2 | up | 2.24 | 1.16 | 4.44E-02 |
| 47 | hsa-miR-17-5p | down | 0.43 | -1.22 | 4.49E-02 |
| 48 | hsa-miR-106a-5p_1ss1AC | down | 0.43 | -1.22 | 4.49E-02 |
| 49 | hsa-mir-4467-p3 | down | 0.25 | -1.99 | 4.56E-02 |
| 50 | hsa-miR-744-5p_R-1 | down | 0.45 | -1.16 | 4.75E-02 |
