# Supplementary material for: Hsa-miR-532-3p protects human decidual mesenchymal stem cells from oxidative stress in recurrent spontaneous abortion via targeting KEAP1
Source: Redox Biol. 2025 Feb 1;80:103508. doi: 10.1016/j.redox.2025.103508 (PMC11847473; doi:10.1016/j.redox.2025.103508)
Supplement: Supplementary Table 4 — Target gene list. [file mmc4.docx]

| Database | Target_gene | Experiment |
| --- | --- | --- |
| mirtarbase | DHCR24 | HITS-CLIP |
| mirtarbase | POLR3G | HITS-CLIP |
| mirtarbase | RAB21 | HITS-CLIP |
| mirtarbase | WT1 | PAR-CLIP |
| mirtarbase | TEAD1 | HITS-CLIP |
| mirtarbase | HEBP2 | HITS-CLIP |
| mirtarbase | STK4 | HITS-CLIP |
| mirtarbase | PWWP2A | HITS-CLIP |
| mirtarbase | PIGR | HITS-CLIP |
| mirtarbase | EZH2 | HITS-CLIP |
| mirtarbase | APP | CLASH |
| mirtarbase | ESM1 | HITS-CLIP |
| mirtarbase | LIF | PAR-CLIP |
| mirtarbase | ATP2B1 | HITS-CLIP |
| mirtarbase | CDK2 | HITS-CLIP |
| mirtarbase | AP2B1 | CLASH |
| mirtarbase | CAMK4 | HITS-CLIP |
| mirtarbase | CAMK2G | HITS-CLIP |
| mirtarbase | CCR4 | HITS-CLIP |
| mirtarbase | CRY2 | CLASH |
| mirtarbase | CTNNB1 | CLASH |
| mirtarbase | DDOST | PAR-CLIP |
| mirtarbase | DDOST | PAR-CLIP |
| mirtarbase | TIMM8A | HITS-CLIP |
| mirtarbase | CELSR3 | HITS-CLIP |
| mirtarbase | ELAVL3 | HITS-CLIP |
| mirtarbase | ELK1 | HITS-CLIP |
| mirtarbase | EREG | PAR-CLIP |
| mirtarbase | F2 | PAR-CLIP |
| mirtarbase | F2 | PAR-CLIP//HITS-CLIP |
| mirtarbase | F2 | HITS-CLIP |
| mirtarbase | GNAS | HITS-CLIP |
| mirtarbase | HAS2 | HITS-CLIP |
| mirtarbase | HTR7 | PAR-CLIP |
| mirtarbase | ILF3 | CLASH |
| mirtarbase | KIFC1 | PAR-CLIP |
| mirtarbase | KIFC1 | PAR-CLIP |
| mirtarbase | KIFC1 | PAR-CLIP |
| mirtarbase | KIFC1 | PAR-CLIP |
| mirtarbase | LTBP2 | HITS-CLIP |
| mirtarbase | MGST3 | HITS-CLIP |
| mirtarbase | NFYA | HITS-CLIP |
| mirtarbase | PAFAH1B1 | HITS-CLIP |
| mirtarbase | PAK3 | HITS-CLIP |
| mirtarbase | PCYT1A | HITS-CLIP |
| mirtarbase | PPM1A | PAR-CLIP |
| mirtarbase | PPP3R1 | CLASH |
| mirtarbase | PPP3R2 | CLASH |
| mirtarbase | PRKAB1 | HITS-CLIP |
| mirtarbase | PRKAR2A | HITS-CLIP |
| mirtarbase | PSMC1 | HITS-CLIP |
| mirtarbase | PTAFR | HITS-CLIP |
| mirtarbase | QSOX1 | HITS-CLIP |
| mirtarbase | PURB | PAR-CLIP |
| mirtarbase | PURB | PAR-CLIP |
| mirtarbase | REL | HITS-CLIP |
| mirtarbase | SCN9A | PAR-CLIP |
| mirtarbase | SCN9A | PAR-CLIP |
| mirtarbase | SCN9A | PAR-CLIP |
| mirtarbase | SCN9A | PAR-CLIP |
| mirtarbase | SREBF1 | HITS-CLIP |
| mirtarbase | TEF | HITS-CLIP |
| mirtarbase | NR2F2 | HITS-CLIP |
| mirtarbase | TRAPPC10 | HITS-CLIP |
| mirtarbase | WNT7B | HITS-CLIP |
| mirtarbase | PXDN | PAR-CLIP |
| mirtarbase | SLC7A5 | PAR-CLIP |
| mirtarbase | SLC7A5 | PAR-CLIP |
| mirtarbase | EIF3I | CLASH |
| mirtarbase | PIAS2 | HITS-CLIP |
| mirtarbase | XPR1 | PAR-CLIP |
| mirtarbase | SLC25A44 | HITS-CLIP |
| mirtarbase | ULK2 | HITS-CLIP |
| mirtarbase | KLHL21 | HITS-CLIP |
| mirtarbase | MFN2 | PAR-CLIP |
| mirtarbase | MFN2 | PAR-CLIP |
| mirtarbase | MFN2 | PAR-CLIP |
| mirtarbase | USP15 | HITS-CLIP |
| mirtarbase | ABCF2 | CLASH |
| mirtarbase | ATP9A | HITS-CLIP |
| mirtarbase | ABI2 | HITS-CLIP |
| mirtarbase | LHFPL2 | HITS-CLIP |
| mirtarbase | CALCOCO2 | HITS-CLIP |
| mirtarbase | KLF2 | HITS-CLIP |
| mirtarbase | TUBB4A | PAR-CLIP |
| mirtarbase | FAM189B | CLASH |
| mirtarbase | FAM189B | CLASH |
| mirtarbase | TRAF3IP2 | PAR-CLIP |
| mirtarbase | TRAF3IP2 | PAR-CLIP |
| mirtarbase | POP4 | HITS-CLIP |
| mirtarbase | ABHD2 | HITS-CLIP |
| mirtarbase | PHLDA1 | PAR-CLIP |
| mirtarbase | SCMH1 | CLASH |
| mirtarbase | CLUAP1 | HITS-CLIP |
| mirtarbase | MRPS27 | PAR-CLIP |
| mirtarbase | ACSL6 | PAR-CLIP |
| mirtarbase | FRAT2 | PAR-CLIP |
| mirtarbase | DNPEP | HITS-CLIP |
| mirtarbase | SMUG1 | PAR-CLIP |
| mirtarbase | TMEM50A | HITS-CLIP |
| mirtarbase | ORC6 | HITS-CLIP |
| mirtarbase | KPNA6 | HITS-CLIP |
| mirtarbase | FGFR1OP2 | HITS-CLIP |
| mirtarbase | TRUB2 | HITS-CLIP |
| mirtarbase | AFF4 | CLASH |
| mirtarbase | VPS4A | PAR-CLIP |
| mirtarbase | IER5 | HITS-CLIP |
| mirtarbase | FAM13B | HITS-CLIP |
| mirtarbase | AMOTL2 | HITS-CLIP |
| mirtarbase | ANKFY1 | HITS-CLIP |
| mirtarbase | RAPGEF6 | HITS-CLIP |
| mirtarbase | MIS18A | PAR-CLIP |
| mirtarbase | CYCS | HITS-CLIP |
| mirtarbase | CYCS | PAR-CLIP |
| mirtarbase | PPP1R12C | HITS-CLIP |
| mirtarbase | CMTM6 | PAR-CLIP |
| mirtarbase | CDCA8 | PAR-CLIP |
| mirtarbase | DARS2 | HITS-CLIP |
| mirtarbase | RBM41 | HITS-CLIP |
| mirtarbase | SYNJ2BP | HITS-CLIP |
| mirtarbase | BCAS4 | PAR-CLIP |
| mirtarbase | EMC7 | HITS-CLIP |
| mirtarbase | DPYSL5 | HITS-CLIP |
| mirtarbase | AGTRAP | HITS-CLIP |
| mirtarbase | THOC2 | HITS-CLIP |
| mirtarbase | GATAD2B | HITS-CLIP |
| mirtarbase | GATAD2B | HITS-CLIP |
| mirtarbase | PHF12 | PAR-CLIP |
| mirtarbase | BCL11B | HITS-CLIP |
| mirtarbase | SH3TC2 | HITS-CLIP |
| mirtarbase | COLGALT1 | CLASH |
| mirtarbase | GSTCD | HITS-CLIP |
| mirtarbase | HSD3B7 | PAR-CLIP |
| mirtarbase | KLHL15 | CLASH |
| mirtarbase | KREMEN1 | HITS-CLIP |
| mirtarbase | FAXC | HITS-CLIP |
| mirtarbase | AIFM2 | HITS-CLIP |
| mirtarbase | ZC3H12C | PAR-CLIP |
| mirtarbase | ZC3H12C | HITS-CLIP |
| mirtarbase | MYLK3 | HITS-CLIP |
| mirtarbase | SYAP1 | HITS-CLIP |
| mirtarbase | MBD6 | PAR-CLIP |
| mirtarbase | PDZD8 | PAR-CLIP |
| mirtarbase | LRRC15 | HITS-CLIP |
| mirtarbase | PTCHD1 | HITS-CLIP |
| mirtarbase | PTCHD1 | HITS-CLIP |
| mirtarbase | THAP6 | PAR-CLIP |
| mirtarbase | RUNDC3B | PAR-CLIP |
| mirtarbase | ZCCHC24 | HITS-CLIP |
| mirtarbase | FOXK1 | HITS-CLIP |
| mirtarbase | SLC25A42 | PAR-CLIP |
| mirtarbase | FAM71F2 | HITS-CLIP |
| mirtarbase | DNAAF3 | HITS-CLIP |
| mirtarbase | PTCHD3 | HITS-CLIP |
| mirtarbase | PTCHD3 | PAR-CLIP |
| mirtarbase | ZNF829 | HITS-CLIP |
| mirtarbase | C18orf32 | PAR-CLIP |
| mirtarbase | ERCC1 | PAR-CLIP |
| mirtarbase | ERCC1 | PAR-CLIP |
| mirtarbase | ERCC1 | HITS-CLIP |
| mirtarbase | CACNA1A | HITS-CLIP |
| mirtarbase | CACNA1A | HITS-CLIP |
| mirtarbase | SLC35E4 | HITS-CLIP |
| mirtarbase | CD200 | HITS-CLIP |
| mirtarbase | GOSR1 | PAR-CLIP |
| mirtarbase | TRPM3 | PAR-CLIP |
| mirtarbase | C2CD4B | PAR-CLIP |
| mirtarbase | TRIM72 | HITS-CLIP |
| mirtarbase | FBXO44 | PAR-CLIP |
| mirtarbase | SBK1 | CLASH |
| mirtarbase | SPAG16 | HITS-CLIP |
| mirtarbase | ZC2HC1C | HITS-CLIP |
| mirtarbase | ZNF682 | HITS-CLIP |
| mirtarbase | PLEKHM3 | HITS-CLIP |
| mirtarbase | TSPAN11 | HITS-CLIP |
| mirtarbase | CLIC5 | HITS-CLIP |
| mirtarbase | RPTN | HITS-CLIP |
| mirtarbase | ADK | HITS-CLIP |
| mirtarbase | MED18 | CLASH |
| mirtarbase | KRBOX4 | PAR-CLIP |
| mirtarbase | CCDC152 | HITS-CLIP |
| mirtarbase | ZNF507 | HITS-CLIP |
| mirtarbase | ZNF844 | PAR-CLIP |
| mirtarbase | TRAPPC3L | HITS-CLIP |
| mirtarbase | CMKLR1 | HITS-CLIP |
| mirtarbase | ANKRD65 | PAR-CLIP |
| mirtarbase | ETV3 | HITS-CLIP |
| mirtarbase | TMEM92 | HITS-CLIP |
| mirtarbase | POLL | HITS-CLIP |
| mirtarbase | BTN3A2 | HITS-CLIP |
| mirtarbase | TBC1D24 | PAR-CLIP |
| mirtarbase | VIPR1 | HITS-CLIP |
| mirtarbase | VIPR1 | HITS-CLIP |
| mirtarbase | RCAN3 | HITS-CLIP |
| mirtarbase | S1PR1 | HITS-CLIP |
| mirtarbase | MAFK | HITS-CLIP |
| mirtarbase | MED14 | CLASH |
| mirtarbase | EPHB2 | HITS-CLIP |
| mirtarbase | PRPF8 | CLASH |
| mirtarbase | TEP1 | PAR-CLIP |
| mirtarbase | TEP1 | PAR-CLIP |
| mirtarbase | PXMP4 | HITS-CLIP |
| mirtarbase | GPR182 | PAR-CLIP |
| mirtarbase | MYO1F | HITS-CLIP |
| mirtarbase | PNMA3 | HITS-CLIP |
| mirtarbase | SVOP | HITS-CLIP |
| mirtarbase | SLCO1B3 | PAR-CLIP |
| mirtarbase | PLXDC1 | PAR-CLIP |
| mirtarbase | C16orf58 | PAR-CLIP |
| mirtarbase | NUDT9 | PAR-CLIP |
| mirtarbase | DDA1 | HITS-CLIP |
| mirtarbase | CEP89 | PAR-CLIP |
| mirtarbase | WDFY2 | HITS-CLIP |
| mirtarbase | ASB16 | PAR-CLIP |
| mirtarbase | ZNF582 | HITS-CLIP |
| mirtarbase | ANKRD18A | HITS-CLIP |
| mirtarbase | BBS5 | PAR-CLIP |
| mirtarbase | NAGS | HITS-CLIP |
| mirtarbase | TRIM35 | HITS-CLIP |
| mirtarbase | USH1G | HITS-CLIP |
| mirtarbase | NEK8 | HITS-CLIP |
| mirtarbase | TMEM119 | PAR-CLIP |
| mirtarbase | TMEM119 | PAR-CLIP |
| mirtarbase | TMEM119 | HITS-CLIP |
| mirtarbase | TMEM119 | PAR-CLIP |
| mirtarbase | SKIDA1 | PAR-CLIP |
| mirtarbase | DNAJB13 | HITS-CLIP |
| mirtarbase | METTL18 | CLASH |
| mirtarbase | LRRC3C | HITS-CLIP |
| mirtarbase | DLEU1 | HITS-CLIP |
| mirtarbase | GRIN2B | HITS-CLIP |
| mirtarbase | GAN | PAR-CLIP |
| mirtarbase | GAN | PAR-CLIP |
| mirtarbase | GAN | PAR-CLIP |
| mirtarbase | GAN | PAR-CLIP |
| mirtarbase | GAN | PAR-CLIP |
| mirtarbase | PTCHD3 | HITS-CLIP |
| mirtarbase | PTCHD3 | PAR-CLIP |
| mirtarbase | PRPF8 | CLASH |
| mirtarbase | SRCIN1 | HITS-CLIP |
| mirtarbase | SRCIN1 | HITS-CLIP |
| mirtarbase | GPAT4 | HITS-CLIP |
| mirtarbase | ZNF878 | HITS-CLIP |
| mirtarbase | ZNF878 | HITS-CLIP |
| mirtarbase | ARSL | HITS-CLIP |
| mirtarbase | MED18 | CLASH |
| mirtarbase | RAB5IF | CLASH |
| mirtarbase | RIPOR2 | HITS-CLIP |
| mirtarbase | GATD3A | HITS-CLIP |
| mirtarbase | PLPBP | PAR-CLIP |
| mirtarbase | RESF1 | HITS-CLIP |
| mirtarbase | MEAK7 | HITS-CLIP |
| mirtarbase | CALHM5 | HITS-CLIP |
| mirtarbase | NOTCH2NLA | HITS-CLIP |
| mirtarbase | NOTCH2NLA | HITS-CLIP |
| mirtarbase | C3orf36 | PAR-CLIP |
| mirtarbase | C3orf36 | PAR-CLIP |
| mirtarbase | C3orf36 | PAR-CLIP |
| mirtarbase | PALM2 | PAR-CLIP |
| mirtarbase | ZNF844 | PAR-CLIP |
| mirtarbase | C2orf48 | HITS-CLIP |
| mirtarbase | PLEKHM3 | HITS-CLIP |
| mirtarbase | TXNRD3NB | HITS-CLIP |
| tarbase | DLL1 | Degradome sequencing |
| tarbase | FBXW11 | Degradome sequencing |
| tarbase | MCL1 | Degradome sequencing//Degradome sequencing//Degradome sequencing |
| tarbase | THBS1 | Degradome sequencing |
| tarbase | DDX5 | Degradome sequencing |
| tarbase | CHSY1 | Degradome sequencing |
| tarbase | MXD4 | Degradome sequencing |
| tarbase | BLCAP | Degradome sequencing |
| tarbase | AXL | Degradome sequencing |
| tarbase | PTPN12 | Degradome sequencing |
| tarbase | TARBP1 | Degradome sequencing |
| tarbase | NFIC | Degradome sequencing |
| tarbase | RBMS1 | Degradome sequencing |
| tarbase | CCND1 | Degradome sequencing//Degradome sequencing |
| tarbase | SLC7A1 | Degradome sequencing |
| tarbase | CDKN1B | Degradome sequencing//Degradome sequencing |
| tarbase | CFL2 | Degradome sequencing//Degradome sequencing//Degradome sequencing |
| tarbase | BACH1 | Degradome sequencing |
| tarbase | SPARC | Degradome sequencing |
| tarbase | FBN1 | Degradome sequencing |
| tarbase | HMGA2 | Degradome sequencing |
| tarbase | RFX4 | Degradome sequencing |
| tarbase | ADCY6 | Degradome sequencing |
| tarbase | SERBP1 | Degradome sequencing |
| tarbase | SFPQ | Degradome sequencing//Degradome sequencing |
| tarbase | TMEM109 | Degradome sequencing |
| tarbase | AK2 | Degradome sequencing |
| tarbase | MED28 | Degradome sequencing |
| tarbase | ANKRD52 | Degradome sequencing |
| tarbase | PRC1 | Degradome sequencing |
| tarbase | HSP90B1 | Degradome sequencing |
| tarbase | PERP | Degradome sequencing//Degradome sequencing |
| tarbase | NOTCH2 | Degradome sequencing//Degradome sequencing |
| tarbase | SOCS1 | Degradome sequencing |
| tarbase | BCL2L2 | Degradome sequencing |
| tarbase | KIF1A | Degradome sequencing |
| tarbase | MET | Degradome sequencing |
| tarbase | EZR | Degradome sequencing//Degradome sequencing//Degradome sequencing |
| tarbase | TP53INP1 | Degradome sequencing |
| tarbase | NFE2L1 | Degradome sequencing |
| tarbase | TGFB1 | Degradome sequencing |
| tarbase | BAX | Degradome sequencing//Degradome sequencing |
| tarbase | AKT2 | Degradome sequencing |
| tarbase | SHC1 | Degradome sequencing |
| tarbase | ATP2B1 | Degradome sequencing |
| tarbase | AGO3 | Degradome sequencing |
| tarbase | SET | Degradome sequencing |
| tarbase | FGFR1 | Degradome sequencing |
| tarbase | TGFBR1 | Degradome sequencing |
| tarbase | BMF | Degradome sequencing |
| tarbase | NLK | Degradome sequencing |
| tarbase | TNPO1 | Degradome sequencing |
| tarbase | ZFP91 | Degradome sequencing |
| tarbase | FOXJ3 | Degradome sequencing |
| tarbase | NCAM1 | Degradome sequencing |
| tarbase | TTYH3 | Degradome sequencing//Degradome sequencing |
| tarbase | EPB41L3 | Degradome sequencing |
| tarbase | ABCF1 | Degradome sequencing |
| tarbase | ACTN1 | Degradome sequencing |
| tarbase | APEH | Degradome sequencing |
| tarbase | XIAP | Degradome sequencing |
| tarbase | ARF6 | Degradome sequencing |
| tarbase | ATP2A2 | Degradome sequencing |
| tarbase | DST | Degradome sequencing |
| tarbase | CD63 | Degradome sequencing |
| tarbase | CDK7 | Degradome sequencing |
| tarbase | CHUK | Degradome sequencing |
| tarbase | AP2S1 | Degradome sequencing//Degradome sequencing//Degradome sequencing |
| tarbase | COL12A1 | Degradome sequencing |
| tarbase | CSNK1A1 | Degradome sequencing |
| tarbase | CUX1 | Degradome sequencing |
| tarbase | CYB561 | Degradome sequencing |
| tarbase | DDOST | Degradome sequencing//Degradome sequencing |
| tarbase | DYNC1H1 | Degradome sequencing |
| tarbase | DSC2 | Degradome sequencing |
| tarbase | RCAN1 | Degradome sequencing |
| tarbase | TSC22D3 | Degradome sequencing |
| tarbase | DUSP1 | Degradome sequencing |
| tarbase | MEGF8 | Degradome sequencing |
| tarbase | EIF4A1 | Degradome sequencing//Degradome sequencing//Degradome sequencing |
| tarbase | EIF4EBP2 | Degradome sequencing |
| tarbase | EIF4G1 | Degradome sequencing |
| tarbase | NR2F6 | Degradome sequencing |
| tarbase | ESRRA | Degradome sequencing |
| tarbase | FGF2 | Degradome sequencing |
| tarbase | GABBR1 | Degradome sequencing |
| tarbase | GLUL | Degradome sequencing |
| tarbase | GNAS | Degradome sequencing |
| tarbase | CXCL3 | Degradome sequencing |
| tarbase | GSK3B | Degradome sequencing |
| tarbase | GTF2A1 | Degradome sequencing |
| tarbase | GTF3C2 | Degradome sequencing |
| tarbase | HTT | Degradome sequencing |
| tarbase | HDGF | Degradome sequencing |
| tarbase | HELLS | Degradome sequencing |
| tarbase | HK1 | Degradome sequencing |
| tarbase | HNRNPD | Degradome sequencing |
| tarbase | HNRNPH1 | Degradome sequencing//Degradome sequencing//Degradome sequencing |
| tarbase | HNRNPL | Degradome sequencing |
| tarbase | HNRNPU | Degradome sequencing |
| tarbase | HOXB3 | Degradome sequencing |
| tarbase | HOXC6 | Degradome sequencing |
| tarbase | IL1A | Degradome sequencing |
| tarbase | INCENP | Degradome sequencing |
| tarbase | INSR | Degradome sequencing |
| tarbase | IREB2 | Degradome sequencing |
| tarbase | ITGA3 | Degradome sequencing |
| tarbase | ITGB1 | Degradome sequencing |
| tarbase | LBR | Degradome sequencing |
| tarbase | LDHB | Degradome sequencing |
| tarbase | LGALS3BP | Degradome sequencing |
| tarbase | MAN2B1 | Degradome sequencing |
| tarbase | MAP1B | Degradome sequencing |
| tarbase | MAZ | Degradome sequencing//Degradome sequencing//Degradome sequencing |
| tarbase | MBD1 | Degradome sequencing |
| tarbase | MBNL1 | Degradome sequencing |
| tarbase | MCM3 | Degradome sequencing |
| tarbase | MDM2 | Degradome sequencing |
| tarbase | KMT2A | Degradome sequencing |
| tarbase | MMP14 | Degradome sequencing |
| tarbase | ABCC1 | Degradome sequencing |
| tarbase | MYBL2 | Degradome sequencing//Degradome sequencing//Degradome sequencing |
| tarbase | NFKB2 | Degradome sequencing |
| tarbase | PCMT1 | Degradome sequencing |
| tarbase | PPM1A | Degradome sequencing |
| tarbase | PPP1CC | Degradome sequencing |
| tarbase | PPP2CB | Degradome sequencing//Degradome sequencing//Degradome sequencing |
| tarbase | PRPS1 | Degradome sequencing |
| tarbase | PTPN4 | Degradome sequencing |
| tarbase | PTPRF | Degradome sequencing |
| tarbase | RAN | Degradome sequencing//Degradome sequencing |
| tarbase | RPL31 | Degradome sequencing |
| tarbase | SIAH1 | Degradome sequencing |
| tarbase | SLC6A9 | Degradome sequencing |
| tarbase | SLC7A2 | Degradome sequencing |
| tarbase | SMARCD2 | Degradome sequencing//Degradome sequencing//Degradome sequencing |
| tarbase | CAPN15 | Degradome sequencing |
| tarbase | SOX11 | Degradome sequencing//Degradome sequencing |
| tarbase | SPTBN1 | Degradome sequencing |
| tarbase | SRF | Degradome sequencing |
| tarbase | SSR3 | Degradome sequencing |
| tarbase | SUPT5H | Degradome sequencing |
| tarbase | TMBIM6 | Degradome sequencing |
| tarbase | TSPYL1 | Degradome sequencing |
| tarbase | TTF1 | Degradome sequencing |
| tarbase | UBE2D3 | Degradome sequencing |
| tarbase | NR1H2 | Degradome sequencing//Degradome sequencing//Degradome sequencing |
| tarbase | XPO1 | Degradome sequencing |
| tarbase | ALMS1 | Degradome sequencing |
| tarbase | ALDH5A1 | Degradome sequencing |
| tarbase | PRRC2A | Degradome sequencing |
| tarbase | KAT6A | Degradome sequencing |
| tarbase | KMT2D | Degradome sequencing |
| tarbase | FZD6 | Degradome sequencing |
| tarbase | HYAL3 | Degradome sequencing |
| tarbase | EEA1 | Degradome sequencing |
| tarbase | STC2 | Degradome sequencing |
| tarbase | SRSF9 | Degradome sequencing |
| tarbase | TNFRSF25 | Degradome sequencing |
| tarbase | EDF1 | Degradome sequencing |
| tarbase | TAX1BP1 | Degradome sequencing |
| tarbase | BAZ1B | Degradome sequencing//Degradome sequencing |
| tarbase | PKMYT1 | Degradome sequencing//Degradome sequencing |
| tarbase | LATS1 | Degradome sequencing |
| tarbase | SLC16A3 | Degradome sequencing |
| tarbase | SMC3 | Degradome sequencing//Degradome sequencing//Degradome sequencing |
| tarbase | MTA2 | Degradome sequencing |
| tarbase | CYTH2 | Degradome sequencing |
| tarbase | B4GALT5 | Degradome sequencing |
| tarbase | EI24 | Degradome sequencing |
| tarbase | EIF5B | Degradome sequencing |
| tarbase | KIAA0100 | Degradome sequencing |
| tarbase | PHACTR2 | Degradome sequencing |
| tarbase | KIAA0232 | Degradome sequencing |
| tarbase | KEAP1 | Degradome sequencing |
| tarbase | TELO2 | Degradome sequencing |
| tarbase | ABCF2 | Degradome sequencing |
| tarbase | ARFRP1 | Degradome sequencing |
| tarbase | ABI2 | Degradome sequencing |
| tarbase | MFSD10 | Degradome sequencing |
| tarbase | ZNF267 | Degradome sequencing |
| tarbase | PCGF3 | Degradome sequencing |
| tarbase | CITED2 | Degradome sequencing |
| tarbase | ZBTB18 | Degradome sequencing |
| tarbase | SLC9A6 | Degradome sequencing |
| tarbase | SYNCRIP | Degradome sequencing |
| tarbase | CCT7 | Degradome sequencing |
| tarbase | FAM189B | Degradome sequencing//Degradome sequencing |
| tarbase | ZNF275 | Degradome sequencing |
| tarbase | PGRMC1 | Degradome sequencing//Degradome sequencing |
| tarbase | EBNA1BP2 | Degradome sequencing |
| tarbase | CCNI | Degradome sequencing |
| tarbase | ILVBL | Degradome sequencing |
| tarbase | RER1 | Degradome sequencing |
| tarbase | ADAMTS5 | Degradome sequencing |
| tarbase | HNRNPUL1 | Degradome sequencing//Degradome sequencing//Degradome sequencing |
| tarbase | HIBADH | Degradome sequencing |
| tarbase | ATXN2L | Degradome sequencing//Degradome sequencing//Degradome sequencing |
| tarbase | KLF8 | Degradome sequencing |
| tarbase | BAHD1 | Degradome sequencing |
| tarbase | BTBD3 | Degradome sequencing |
| tarbase | SACM1L | Degradome sequencing |
| tarbase | TBC1D2B | Degradome sequencing |
| tarbase | KDM6B | Degradome sequencing |
| tarbase | NCDN | Degradome sequencing |
| tarbase | SNX13 | Degradome sequencing |
| tarbase | LARP4B | Degradome sequencing |
| tarbase | ARL6IP1 | Degradome sequencing |
| tarbase | SYNE2 | Degradome sequencing//Degradome sequencing |
| tarbase | KHNYN | Degradome sequencing |
| tarbase | ADNP | Degradome sequencing |
| tarbase | CTDNEP1 | Degradome sequencing |
| tarbase | CBX6 | Degradome sequencing |
| tarbase | SRRM2 | Degradome sequencing//Degradome sequencing//Degradome sequencing |
| tarbase | TNPO3 | Degradome sequencing |
| tarbase | CARHSP1 | Degradome sequencing |
| tarbase | MTCH1 | Degradome sequencing |
| tarbase | CNOT10 | Degradome sequencing |
| tarbase | KBTBD2 | Degradome sequencing |
| tarbase | ZNF451 | Degradome sequencing |
| tarbase | GNL3 | Degradome sequencing |
| tarbase | MYOF | Degradome sequencing |
| tarbase | PELP1 | Degradome sequencing |
| tarbase | AFF4 | Degradome sequencing |
| tarbase | ATAD2 | Degradome sequencing |
| tarbase | MED4 | Degradome sequencing |
| tarbase | CNIH4 | Degradome sequencing |
| tarbase | CERS2 | Degradome sequencing |
| tarbase | TNPO2 | Degradome sequencing |
| tarbase | VILL | Degradome sequencing |
| tarbase | VPS36 | Degradome sequencing |
| tarbase | DCTN4 | Degradome sequencing |
| tarbase | ERGIC2 | Degradome sequencing |
| tarbase | SUCO | Degradome sequencing |
| tarbase | SFMBT1 | Degradome sequencing |
| tarbase | DPH5 | Degradome sequencing//Degradome sequencing |
| tarbase | SLC25A39 | Degradome sequencing |
| tarbase | BCL11A | Degradome sequencing |
| tarbase | PPP1R12C | Degradome sequencing//Degradome sequencing//Degradome sequencing//Degradome sequencing |
| tarbase | NCAPG2 | Degradome sequencing |
| tarbase | CMTM6 | Degradome sequencing |
| tarbase | FAM118A | Degradome sequencing |
| tarbase | DET1 | Degradome sequencing |
| tarbase | ZDHHC4 | Degradome sequencing |
| tarbase | PBRM1 | Degradome sequencing |
| tarbase | UBE2W | Degradome sequencing |
| tarbase | SYNJ2BP | Degradome sequencing |
| tarbase | YOD1 | Degradome sequencing |
| tarbase | RBM22 | Degradome sequencing |
| tarbase | TMEM30A | Degradome sequencing |
| tarbase | ASH1L | Degradome sequencing |
| tarbase | RCC2 | Degradome sequencing |
| tarbase | NSFL1C | Degradome sequencing |
| tarbase | MEPCE | Degradome sequencing |
| tarbase | TM9SF3 | Degradome sequencing |
| tarbase | STARD7 | Degradome sequencing |
| tarbase | RGMA | Degradome sequencing |
| tarbase | CCDC47 | Degradome sequencing |
| tarbase | ACKR3 | Degradome sequencing |
| tarbase | FAM219B | Degradome sequencing |
| tarbase | THOC2 | Degradome sequencing |
| tarbase | GATAD2B | Degradome sequencing//Degradome sequencing |
| tarbase | ESYT2 | Degradome sequencing |
| tarbase | NUFIP2 | Degradome sequencing |
| tarbase | KIAA1328 | Degradome sequencing |
| tarbase | ZBTB4 | Degradome sequencing |
| tarbase | CHD8 | Degradome sequencing |
| tarbase | CREBZF | Degradome sequencing |
| tarbase | KMT2C | Degradome sequencing |
| tarbase | ENPP5 | Degradome sequencing |
| tarbase | SNX16 | Degradome sequencing |
| tarbase | GIGYF1 | Degradome sequencing//Degradome sequencing |
| tarbase | NUCKS1 | Degradome sequencing |
| tarbase | CERK | Degradome sequencing |
| tarbase | BCL11B | Degradome sequencing |
| tarbase | MARCKSL1 | Degradome sequencing |
| tarbase | MBOAT7 | Degradome sequencing |
| tarbase | ATP13A3 | Degradome sequencing |
| tarbase | COLGALT1 | Degradome sequencing |
| tarbase | CPSF7 | Degradome sequencing//Degradome sequencing |
| tarbase | KIAA0319L | Degradome sequencing |
| tarbase | GPR157 | Degradome sequencing |
| tarbase | TRABD | Degradome sequencing |
| tarbase | PUS1 | Degradome sequencing |
| tarbase | ANP32E | Degradome sequencing |
| tarbase | SLC25A28 | Degradome sequencing//Degradome sequencing//Degradome sequencing |
| tarbase | SESN2 | Degradome sequencing//Degradome sequencing |
| tarbase | TNRC18 | Degradome sequencing |
| tarbase | TUBA1C | Degradome sequencing |
| tarbase | TNKS1BP1 | Degradome sequencing |
| tarbase | ZC3H12C | Degradome sequencing |
| tarbase | MIDN | Degradome sequencing//Degradome sequencing |
| tarbase | LMF2 | Degradome sequencing//Degradome sequencing//Degradome sequencing |
| tarbase | OXNAD1 | Degradome sequencing |
| tarbase | ZBTB47 | Degradome sequencing//Degradome sequencing//Degradome sequencing |
| tarbase | PHF21B | Degradome sequencing |
| tarbase | SLC2A13 | Degradome sequencing//Degradome sequencing//Degradome sequencing//Degradome sequencing |
| tarbase | PDZD8 | Degradome sequencing |
| tarbase | NIPA1 | Degradome sequencing |
| tarbase | NACC2 | Degradome sequencing |
| tarbase | SOGA1 | Degradome sequencing |
| tarbase | STK35 | Degradome sequencing//Degradome sequencing |
| tarbase | CACUL1 | Degradome sequencing |
| tarbase | SESN3 | Degradome sequencing |
| tarbase | TMTC2 | Degradome sequencing |
| tarbase | QSOX2 | Degradome sequencing//Degradome sequencing |
| tarbase | ZNF367 | Degradome sequencing//Degradome sequencing//Degradome sequencing |
| tarbase | VMA21 | Degradome sequencing |
| tarbase | UNC5B | Degradome sequencing |
| tarbase | TPCN2 | Degradome sequencing |
| tarbase | FAM171A1 | Degradome sequencing |
| tarbase | HNRNPUL2 | Degradome sequencing//Degradome sequencing |
| tarbase | FOXK1 | Degradome sequencing |
| tarbase | TMED4 | Degradome sequencing |
| tarbase | PRR14L | Degradome sequencing |
| tarbase | LCLAT1 | Degradome sequencing |
| tarbase | RASSF3 | Degradome sequencing |
| tarbase | ZNF776 | Degradome sequencing |
| tarbase | XKR6 | Degradome sequencing |
| tarbase | RBM12B | Degradome sequencing |
| tarbase | SNX19 | Degradome sequencing |
| tarbase | ARHGAP27 | Degradome sequencing |
| tarbase | PEG10 | Degradome sequencing |
| tarbase | ERCC1 | Degradome sequencing |
| tarbase | MTR | Degradome sequencing |
| tarbase | CD4 | Degradome sequencing |
| tarbase | CSF3 | Degradome sequencing |
| tarbase | EDA | Degradome sequencing |
| tarbase | FPR2 | Degradome sequencing |
| tarbase | SPOP | Degradome sequencing |
| tarbase | IBA57 | Degradome sequencing |
| tarbase | AFMID | Degradome sequencing |
| tarbase | KRIT1 | Degradome sequencing//Degradome sequencing |
| tarbase | DCAF6 | Degradome sequencing |
| tarbase | CANX | Degradome sequencing//Degradome sequencing//Degradome sequencing |
| tarbase | CELF1 | Degradome sequencing//Degradome sequencing |
| tarbase | HNRNPH2 | Degradome sequencing |
| tarbase | UNKL | Degradome sequencing |
| tarbase | PICK1 | Degradome sequencing |
| tarbase | STRN4 | Degradome sequencing |
| tarbase | PPAN-P2RY11 | Degradome sequencing |
| tarbase | CEP170 | Degradome sequencing |
| tarbase | TTC39A | Degradome sequencing |
| tarbase | ACLY | Degradome sequencing |
| tarbase | ACO2 | Degradome sequencing |
| tarbase | FBXL19 | Degradome sequencing//Degradome sequencing |
| tarbase | C6orf136 | Degradome sequencing//Degradome sequencing |
| tarbase | NHLH2 | Degradome sequencing |
| tarbase | ZNF276 | Degradome sequencing |
| tarbase | CNBP | Degradome sequencing |
| tarbase | HMOX2 | Degradome sequencing |
| tarbase | MAMSTR | Degradome sequencing |
| tarbase | EVA1A | Degradome sequencing |
| tarbase | IGSF9 | Degradome sequencing |
| tarbase | ATXN7L3B | Degradome sequencing |
| tarbase | MGRN1 | Degradome sequencing |
| tarbase | GPSM1 | Degradome sequencing |
| tarbase | CAND2 | Degradome sequencing |
| tarbase | SLC37A4 | Degradome sequencing |
| tarbase | SBNO1 | Degradome sequencing |
| tarbase | CAMTA2 | Degradome sequencing |
| tarbase | TECPR2 | Degradome sequencing |
| tarbase | RABL6 | Degradome sequencing |
| tarbase | SCN8A | Degradome sequencing |
| tarbase | UCKL1 | Degradome sequencing//Degradome sequencing//Degradome sequencing |
| tarbase | SMAP2 | Degradome sequencing |
| tarbase | ZNF615 | Degradome sequencing |
| tarbase | ZNF497 | Degradome sequencing |
| tarbase | CFD | Degradome sequencing//Degradome sequencing |
| tarbase | DLX4 | Degradome sequencing |
| tarbase | KCNG1 | Degradome sequencing |
| tarbase | MAFK | Degradome sequencing |
| tarbase | PBX2 | Degradome sequencing |
| tarbase | POLB | Degradome sequencing |
| tarbase | SLC9A2 | Degradome sequencing |
| tarbase | PPFIBP2 | Degradome sequencing |
| tarbase | PPFIA1 | Degradome sequencing |
| tarbase | RFXANK | Degradome sequencing |
| tarbase | STK19 | Degradome sequencing |
| tarbase | ARHGAP35 | Degradome sequencing |
| tarbase | PABPN1 | Degradome sequencing//Degradome sequencing |
| tarbase | IER2 | Degradome sequencing//Degradome sequencing |
| tarbase | FAT1 | Degradome sequencing |
| tarbase | MLF2 | Degradome sequencing |
| tarbase | ALDH6A1 | Degradome sequencing |
| tarbase | TRIM28 | Degradome sequencing |
| tarbase | POU2AF1 | Degradome sequencing |
| tarbase | STK25 | Degradome sequencing |
| tarbase | PRPF8 | Degradome sequencing//Degradome sequencing//Degradome sequencing |
| tarbase | HEXIM1 | Degradome sequencing//Degradome sequencing |
| tarbase | SRCAP | Degradome sequencing |
| tarbase | SOX12 | Degradome sequencing |
| tarbase | KIF3A | Degradome sequencing |
| tarbase | DDX20 | Degradome sequencing |
| tarbase | FBXW8 | Degradome sequencing |
| tarbase | HNRNPH3 | Degradome sequencing//Degradome sequencing |
| tarbase | PHLDA3 | Degradome sequencing |
| tarbase | ETHE1 | Degradome sequencing//Degradome sequencing//Degradome sequencing//Degradome sequencing |
| tarbase | PATZ1 | Degradome sequencing |
| tarbase | ZNF324 | Degradome sequencing |
| tarbase | LRIG2 | Degradome sequencing//Degradome sequencing |
| tarbase | KLHDC10 | Degradome sequencing |
| tarbase | CIC | Degradome sequencing |
| tarbase | PCF11 | Degradome sequencing//Degradome sequencing |
| tarbase | TRAPPC4 | Degradome sequencing |
| tarbase | LSM7 | Degradome sequencing |
| tarbase | TMEM9 | Degradome sequencing |
| tarbase | ZC3HC1 | Degradome sequencing |
| tarbase | TDP2 | Degradome sequencing |
| tarbase | WAC | Degradome sequencing |
| tarbase | SIX2 | Degradome sequencing |
| tarbase | CPSF2 | Degradome sequencing |
| tarbase | INO80 | Degradome sequencing |
| tarbase | OTUB1 | Degradome sequencing |
| tarbase | PACS1 | Degradome sequencing |
| tarbase | SAMD4B | Degradome sequencing |
| tarbase | ARFGAP1 | Degradome sequencing |
| tarbase | TBC1D16 | Degradome sequencing |
| tarbase | YLPM1 | Degradome sequencing |
| tarbase | NLGN2 | Degradome sequencing |
| tarbase | MYL6 | Degradome sequencing |
| tarbase | VPS11 | Degradome sequencing |
| tarbase | NSD1 | Degradome sequencing//Degradome sequencing |
| tarbase | EXO5 | Degradome sequencing |
| tarbase | HOXB4 | Degradome sequencing |
| tarbase | PHF23 | Degradome sequencing//Degradome sequencing//Degradome sequencing |
| tarbase | OGFOD3 | Degradome sequencing |
| tarbase | ZNF768 | Degradome sequencing//Degradome sequencing |
| tarbase | TM2D3 | Degradome sequencing//Degradome sequencing |
| tarbase | RAI1 | Degradome sequencing//Degradome sequencing |
| tarbase | SNX27 | Degradome sequencing//Degradome sequencing |
| tarbase | TM2D1 | Degradome sequencing//Degradome sequencing |
| tarbase | ZDHHC16 | Degradome sequencing |
| tarbase | BRSK1 | Degradome sequencing |
| tarbase | ZNF514 | Degradome sequencing |
| tarbase | SYDE1 | Degradome sequencing |
| tarbase | TRIM41 | Degradome sequencing |
| tarbase | CCDC97 | Degradome sequencing |
| tarbase | STX1B | Degradome sequencing |
| tarbase | LENG8 | Degradome sequencing |
| tarbase | C21orf58 | Degradome sequencing |
| tarbase | KCNE4 | Degradome sequencing |
| tarbase | KIAA2013 | Degradome sequencing |
| tarbase | SPPL3 | Degradome sequencing |
| tarbase | TRIM11 | Degradome sequencing |
| tarbase | PRRT2 | Degradome sequencing//Degradome sequencing |
| tarbase | MMP21 | Degradome sequencing |
| tarbase | SDE2 | Degradome sequencing |
| tarbase | PHYKPL | Degradome sequencing |
| tarbase | NEK8 | Degradome sequencing |
| tarbase | NAT8L | Degradome sequencing |
| tarbase | ISCA2 | Degradome sequencing |
| tarbase | FAM43B | Degradome sequencing |
| tarbase | EEF1D | Degradome sequencing//Degradome sequencing |
| tarbase | WDR13 | Degradome sequencing |
| tarbase | CCIN | Degradome sequencing |
| tarbase | ACAP1 | Degradome sequencing |
| tarbase | RPL36 | Degradome sequencing//Degradome sequencing |
| tarbase | INO80E | Degradome sequencing |
| tarbase | C19orf48 | Degradome sequencing |
| tarbase | CCDC9 | Degradome sequencing |
| tarbase | RABAC1 | Degradome sequencing |
| tarbase | OXSM | Degradome sequencing |
| tarbase | EME2 | Degradome sequencing |
| tarbase | IRF3 | Degradome sequencing |
| tarbase | RPS4X | Degradome sequencing |
| tarbase | ZBTB48 | Degradome sequencing |
| tarbase | IRS4 | Degradome sequencing |
| tarbase | MYL5 | Degradome sequencing |
| tarbase | PFKL | Degradome sequencing |
| tarbase | GAN | Degradome sequencing//Degradome sequencing |
| tarbase | DYNLL2 | Degradome sequencing//Degradome sequencing//Degradome sequencing//Degradome sequencing |
| tarbase | SPPL2B | Degradome sequencing |
| tarbase | RAB29 | Degradome sequencing |
| tarbase | SRPRA | Degradome sequencing |
| tarbase | CNOT9 | Degradome sequencing//Degradome sequencing |
| tarbase | PIP4K2B | Degradome sequencing |
| tarbase | MELTF | Degradome sequencing |
| tarbase | MLLT6 | Degradome sequencing |
| tarbase | TXNIP | Degradome sequencing//Degradome sequencing//Degradome sequencing |
| tarbase | BEX3 | Degradome sequencing |
| tarbase | CEP126 | Degradome sequencing |
| tarbase | PGGHG | Degradome sequencing |
| tarbase | PCNX3 | Degradome sequencing |
| tarbase | ZCCHC3 | Degradome sequencing |
| tarbase | ADGRA3 | Degradome sequencing |
| tarbase | PIDD1 | Degradome sequencing |
| tarbase | MTURN | Degradome sequencing |
| tarbase | NSD2 | Degradome sequencing |
| tarbase | INTS14 | Degradome sequencing |
| tarbase | JPT1 | Degradome sequencing |
| tarbase | DENND11 | Degradome sequencing |
| tarbase | SEPTIN8 | Degradome sequencing |
| tarbase | TMEM250 | Degradome sequencing//Degradome sequencing |
| tarbase | DARS1 | Degradome sequencing |
| tarbase | RETREG2 | Degradome sequencing |
| tarbase | CCN2 | Degradome sequencing |
| tarbase | H2AX | Degradome sequencing//Degradome sequencing |
| tarbase | CAVIN1 | Degradome sequencing |
| tarbase | PNMA8B | Degradome sequencing |
| tarbase | PGAP6 | Degradome sequencing |
| tarbase | RTL10 | Degradome sequencing |
| tarbase | JPT2 | Degradome sequencing//Degradome sequencing |
| tarbase | DIPK1B | Degradome sequencing |
| tarbase | AC022966.1 | Degradome sequencing |
| tarbase | BLOC1S5-TXNDC5 | Degradome sequencing//Degradome sequencing |
