# Supplementary material for: Hsa-miR-532-3p protects human decidual mesenchymal stem cells from oxidative stress in recurrent spontaneous abortion via targeting KEAP1
Source: Redox Biol. 2025 Feb 1;80:103508. doi: 10.1016/j.redox.2025.103508 (PMC11847473; doi:10.1016/j.redox.2025.103508)
Supplement: Supplementary Table 5 — Information of sample providers. [file mmc5.docx]

Supplementary Table 5: Information of sample providers

| No. | Group | Age | BMI | Gestational week | Gestation | Parturition | Times of spontaneous abortions |  |
| --- | --- | --- | --- | --- | --- | --- | --- | --- |
| 1 | Control | 27 | 30.1 | 11 | 0 | 0 | 1 |  |
| 2 | Control | 28 | 28.8 | 8 | 0 | 0 | 1 |  |
| 3 | Control | 29 | 33.5 | 9 | 0 | 0 | 1 |  |
| 4 | Control | 31 | 30.8 | 10 | 0 | 0 | 1 |  |
| 5 | Control | 26 | 26.1 | 9 | 0 | 0 | 1 |  |
| 6 | RSA | 26 | 27.3 | 8 | 3 | 0 | 3 |  |
| 7 | RSA | 31 | 33.2 | 10 | 4 | 0 | 4 |  |
| 8 | RSA | 27 | 28.4 | 9 | 3 | 0 | 3 |  |
| 9 | RSA | 28 | 30.3 | 7 | 3 | 0 | 3 |  |
| 10 | RSA | 30 | 31.1 | 8 | 4 | 0 | 4 |  |
