# Supplementary material for: Hsa-miR-532-3p protects human decidual mesenchymal stem cells from oxidative stress in recurrent spontaneous abortion via targeting KEAP1
Source: Redox Biol. 2025 Feb 1;80:103508. doi: 10.1016/j.redox.2025.103508 (PMC11847473; doi:10.1016/j.redox.2025.103508)
Supplement: Multimedia component 11 [file mmc11.pdf]

ARRIVE 指南

Study Design

This study aimed to investigate the role of miR-532-3p in a mouse model of recurrent spontaneous abortion (RSA). Thirty female CBA/J mice were randomly assigned to three groups: RSA model with miRNA intervention, RSA model with negative control, and a normal control group. The experimental design assessed the effects of miRNA modulation on pregnancy outcomes and oxidative stress in decidual tissues.

Sample Size

A total of thirty female CBA/J mice were used, with ten mice in each group (normal control, RSA agomir NC, and RSA agomiR-532-3p). The sample size was based on previous studies and preliminary calculations to ensure statistical power for detecting differences in pregnancy outcomes and oxidative stress markers.

Inclusion and Exclusion Criteria

Female CBA/J mice of similar age and weight were included to minimize variability. Mice with irregular mating behaviors or failure to demonstrate pregnancy were excluded. Non-pregnant mice were also excluded from further analysis to ensure valid pregnancy data.

Randomization

Mice were randomly assigned to one of the three experimental groups using a random number generator to prevent bias in group allocation. Randomization helped ensure that any observed differences were due to the miRNA intervention, not pre-existing group differences.

Blinding

The investigators performing outcome assessments, including measurements of embryo resorption rates, tissue morphology, and protein expression, were blinded to group allocations to minimize bias and ensure objective data collection.

Outcome Measures

Key outcome measures included:

Pregnancy outcomes: Abortion rate, embryo resorption rate, and fetal survival rate.

Decidual tissue morphology: Histological examination via Hematoxylin and Eosin (HE) staining.

Oxidative stress markers: Protein expression levels of KEAP1 and other related markers.

Gene expression: qRT-PCR analysis of relevant target genes.

Cell apoptosis: Flow cytometry analysis of apoptosis in uterine decidual tissue.

Statistical Methods

Data were analyzed using GraphPad Prism software. One-way ANOVA followed by Tukey's post-hoc test was used for normally distributed data. Non-parametric tests (e.g., Mann-Whitney U test) were used for non-normally distributed data. A p-value of < 0.05 was considered statistically significant. Statistical power was calculated based on previous literature.

Experimental Animals

Thirty female CBA/J mice were obtained from the Laboratory Animal Center of Jinan University. Mice were housed under standard laboratory conditions with a 12-hour light/dark cycle and provided food and water ad libitum. They were acclimatized for one week before experimentation.

Experimental Procedures

All experiments were approved by the Laboratory Animal Ethics Committee of Jinan University (IACUC-20201126-05). Mice were randomly divided into three groups. Twenty female CBA/J mice were co-housed with five male DBA/2J mice to establish the RSA model, and another 10 were co-housed with 5 male BALB/c mice to establish a normal pregnancy model. The mice were injected with agomir NC or agomiR-532-3p at 10 nmol per mouse in 200 μl saline via tail vein injection. Starting from day 0, saline solution was administered every 3 days. The mice were sacrificed on day 11.5 for further analysis of embryo resorption rates, KEAP1 expression, and oxidative stress markers.

Results

In the RSA model, miR-532-3p expression was significantly decreased in decidual tissues, while overexpression of miR-532-3p in the RSA-agomir-532-3p group significantly reduced the abortion rate and KEAP1 expression. The overexpression also increased oxidative stress markers, such as T-AOC, SOD, GSH-PX, CAT, and GSH/GSSG ratios, indicating reduced oxidative damage and improved pregnancy outcomes.
